# Temporal evolution of insecticide resistance and bionomics in *Anopheles funestus,* a key malaria vector in Uganda

**DOI:** 10.1038/s41598-024-83689-6

**Published:** 2024-12-30

**Authors:** Ambrose Oruni, Magellan Tchouakui, Carlos S. Djoko Tagne, Jack Hearn, Jonathan Kayondo, Charles S. Wondji

**Affiliations:** 1 Entomology Department, Uganda Virus Research Institute, P.O. BOX 49, Entebbe, Uganda; 2 Centre for Research in Infectious Diseases, LSTM-Research Unit, P.O BOX 3591, Yaoundé, Cameroon; 3 Vector Biology Department, Liverpool School of Tropical Medicine, Liverpool, L3 5QA UK; 4 Centre for Epidemiology and Planetary Health, Scotland’s Rural College (SRUC), Inverness, IV2 5NA UK; 5 International Institute of Tropical Agriculture (IITA), P.O. Box 2008, Yaoundé, Cameroon

**Keywords:** Insecticide resistance, *Anopheles funestus*, Genetic markers, Temporal, Phenotypes, Uganda, Biological techniques, Ecology, Genetics, Zoology

## Abstract

Insecticide resistance escalation is decreasing the efficacy of vector control tools. Monitoring vector resistance is paramount in order to understand its evolution and devise effective counter-solutions. In this study, we monitored insecticide resistance patterns, vector population bionomics and genetic variants associated with resistance over 3 years from 2021 to 2023 in Uganda. *Anopheles funestus s.s* was the predominant species in Mayuge but with evidence of hybridization with other species of the *An. funestus* group. Sporozoite infection rates were relatively very high with a peak of 20.41% in March 2022. Intense pyrethroid resistance was seen against pyrethroids up to 10-times the diagnostic concentration but partial recovery of susceptibility in PBO synergistic assays. Among bednets, only PBO-based nets (PermaNet 3.0 Top and Olyset Plus) and chlorfenapyr-based net (Interceptor G2) had high mortality rates. Mosquitoes were fully susceptible to chlorfenapyr and organophosphates, moderately resistant to clothianidin and resistant to carbamates. The allele frequency of key P450, CYP9K1, resistance marker was constantly very high but that for CYP6P9A/b were very low. Interestingly, we report the first detection of resistance alleles for Ace1 gene (RS =  ~ 13%) and Rdl gene (RS =  ~ 21%, RR =  ~ 4%) in Uganda. The qRT-PCR revealed that Cytochrome P450s CYP9K1, CYP6P9A, CYP6P9b, CYP6P5 and CYP6M7 were consistently upregulated while a glutathione-S-transferase gene (GSTE2) showed low expression. Our study shows the complexity of insecticide resistance patterns and underlying mechanisms, hence constant and consistent spatial and temporal monitoring is crucial to rapidly detect changing resistance profiles which is key in informing deployment of counter interventions.

## Introduction

Malaria remains the largest contributor to mortality and morbidity especially in African pregnant women and children. In 2022, there were an estimated 249 million cases globally with an estimated 608,000 deaths with the majority occurring in Sub-Saharan Africa mainly due to *P. falciparum* parasite^1^. Uganda underwent a malaria resurgence^2^ and was among the four countries that contributed to almost 50% of the global cases^1^. Despite the deployment of various tools such as; Long Lasting Insecticidal Nets (LLINs), Indoor residual Spraying (IRS) and larvicides, progress has stalled. Vector control is currently the pillar of malaria control targeting mainly *Anopheles funestus* and *Anopheles gambiae* species and solely rely on the use of insecticides^3^. Pyrethroids were the only class of insecticides used in bednets—the primary malaria control tool in Africa^1^. As a result, pyrethroid resistance became widespread in major malaria vectors and currently, there is now resistance to all classes of insecticides including the newly introduced ones^4–6^. The scale-up of vector control interventions especially bednets could have contributed to the spread of resistance due to the selection pressure on an evolutionary scale. By 2020, bednet use in Africa in some countries like Uganda reached the 80% mark needed for universal coverage, with others following a similar trend^7^. The efficacy of bednets especially single active ingredient (AI) decreased over the years due to pyrethroid resistance^8^ as seen in Uganda^9,10^, Malawi^11^ and Cameroon^12^. To manage insecticide resistance, formerly, piperonyl butoxide (PBO) was incorporated into LLINs to increase the potency of pyrethroids hence increasing the killing effect^13^. Indeed, PBO nets produced a significantly better performance than pyrethroid-only bednets for example in Tanzania^14^ and Uganda^15,16^ but the effectiveness has also decreased in recent years^17–19^. A key factor decreasing the efficacy of PBO nets is the rise of resistance escalation as reported in Uganda^20^, Malawi^11^ and Ghana^21^ even further leading to cross-resistance with other classes of insecticides such as carbamates^22^. These scenarios underscore the importance of probably avoiding continuous deployment of the same classes of insecticides in areas with escalated resistance levels or cross-resistance. Hence, the recent strategy has been the introduction of two new classes of insecticides; Pyrroles for bednets and Neonicotinoids for IRS^23^ and these are currently showing very promising results due to different modes of action. Clothianidin is a neonicotinoid that acts by targeting the nicotinic acetylcholine receptor (nAChR) in the insect central nervous system^24^. Chlorfenapyr is a pyrrole which acts by disrupting energy production using toxins produced by oxidases in insect cells^25,26^.

In *An. funestus*, insecticide resistance is principally driven by the overexpression of metabolic genes, mainly cytochrome P450s (CYPs)^27–29^ such as; CYP6P9A and CYP6P9b^30^ and CYP9K1 which was discovered to be overexpressed in Uganda and Cameroon^31,32^. The expression of these key metabolic genes significantly differs geographically in *Anopheles* vectors^33^. These variations indicate a possible existence of distinct selection pressures and *de-novo* evolutionary processes with limited gene flow over long distances possibly due to geographical barriers or just insufficient time for resistance alleles to spread between regions of Africa. Furthermore, non-synonymous single nucleotide polymorphisms (SNPs) alleles have been detected in CYP6P9A^34^, CYP6P9b^35^ and CYP9K1^36^ and also two structural variants (SVs); the 6.5-Kb SV found between CYP6P9A and CYP6P9b genes^37^ and 4.3-Kb SV which is found between CYP6P9b and CYP6P5 genes^38^. The genetic haplotype of the 6.5-Kb SV is closely linked with *CYP6P9a/b* markers and for 4.3-Kb SV with *CYP9K1* marker hence having the same allele frequency pattern as the metabolic markers and together, they drastically reduce the performance of bednets^38^.

Monitoring Insecticide resistance in principal malaria vectors is a key pillar of the Global Plan for insecticide resistance management^39^. This undertaking usually assists in uncovering any changes and signals of selection, resistance escalation and spread, which is critical in informing timely and counter-effective solutions. Both insecticide susceptibility tests and use of resistance markers are applied in monitoring resistance^39,40^. This has helped uncover key information on the evolution of resistance geographically like the distribution of resistance markers such as; *CYP6P9a* and *CYP6P9b* being fixed in southern Africa^34,35^ and southern Tanzania^41^, but at very low allele frequencies in Uganda^9^. In contrast, the *CYP9K1* marker (*G454A*) being fixed in Uganda and parts of Cameroon but very low frequencies in southern Africa^9,36^. Furthermore, other known resistance markers discovered in *An. funestus* include; a SNP found within glutathione-s-transferase epsilon 2 (GSTE2) gene, *GSTe2-119F* metabolic marker discovered in West Africa at high frequency^42^ but still at very low frequency in East Africa^9,41^ and two target-site markers; in the acetylcholinesterase (Ace1) gene, *N458I-Ace1* discovered in Malawi which confer resistance to organophosphates and carbamates^43^ and in the dieldrin resistance gene (Rdl), *A296S-Rdl* discovered in Burkina-Faso and Cameroon conferring resistance to dieldrin and DDT^44^. Except for *A296S* which was detected in 2009^44^ but subsequently reduced in frequency to zero by 2020^9^, mutant alleles for both target site markers have never been detected in East Africa. The only known target-site marker is the *Vgsc-L976F* variant which is for a *knockdown resistance* (*kdr*), was detected very recently in East Africa and confers resistance to DDT but is currently localised in Tanzania^45^.

Insecticide resistance is very dynamic in space and time. Mosquito populations often change depending on selection pressure which eventually could alter resistance mechanisms and population structure as seen with the rise in *An. funestus* populations due to LLIN use^46^. In 2020, pyrethroid resistance escalation was detected in Uganda but the markers did not associate with the resistance phenotype despite the overexpression of some key genes^9^. We carried out a 3-year temporal study from 2021 to; (1) assess if these patterns were consistent, (2) elucidate the genetic variants that could be driving pyrethroid resistance in Uganda and (3) document temporal changes in vector bionomics. This information is crucial for in-country malaria control efforts, especially at a time when a variety of interventions are being rolled out and their impact needs to be accurately assessed.

## Methods

### Ethical statement

The protocol to conduct this study including Material Transfer Agreement (MTA) was approved by The Uganda Virus Research Institute Research Ethics Committee (UVRI REC) (Ref: GC/127/833) and the Uganda National Council for Science and Technology (UNCST) (HS2063ES). Before mosquito collections, written, informed and signed consents were obtained from the heads of the households. All information regarding the study was included in the consent form and participants were taken through the consent forms where needed. Additionally, the information in the consent form was translated into the local language where needed.

### Study site and mosquito collections

**Fig. 1:**
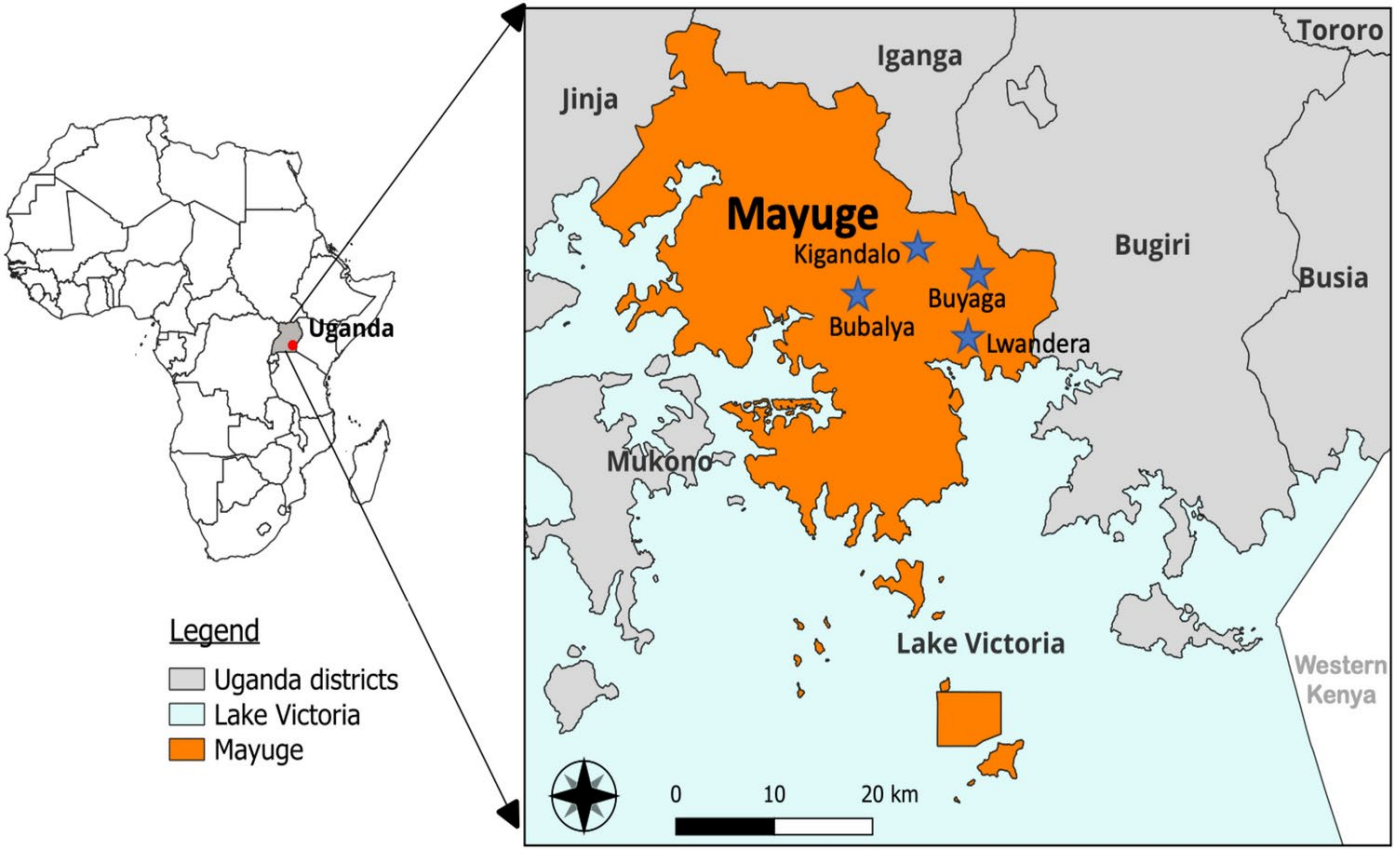
Map of mosquito collection sites within Mayuge district. Mayuge district is demarcated from other neighbouring districts by brown colour. The villages where households were recruited are marked with a blue star. The map was generated using free and open source QGIS software version 3.38.

The study was conducted in Mayuge district in eastern Uganda (00° 24ʹ 20.06ʺ N 033° 47ʹ 57.50ʺ E) (Fig. 1) from 2021 to 2023 targeting four villages; Kigandalo, Buyaga, Bubalya (Kigulu) and Lwandera (Bukabooli) (Fig. 1). Mayuge is not an IRS district i.e. household spray operations are not conducted in the district as part of the national malaria control program (NMCP) yearly activities, but, it immediately borders Bugiri and is close to Tororo which are all IRS districts. History of IRS include usage of bendiocarb and Actellic interchangeably up to 2018, then Fludora Fusion and Sumishield from 2019-2022 and back to Actellic in 2023 and 2024 with few houses receiving Sumishield in 2023^2^. Vector control interventions in Mayuge have mainly been through the distribution of ITNs since 2012^47^. In our study, mosquitoes were collected in October 2021, March 2022, November 2022, May 2023 and November 2023 from 50 households selected at random. The electric prokopack aspiration technique (John W. Hock co., USA) was used since the aim was to collect alive blood-fed indoor resting female mosquitoes. Mosquito collection was done for five days in the early morning between 4.30 and 9 a.m. Aspirated mosquitoes were kept in cages and then morphologically identified using keys by Coetzee^48^ and separated into *Anopheles gambiae s.l* from *An. funestus s.l*, other Culicines and male categories. The sorted *Anopheles* mosquitoes were then transferred to paper cups (separating *An. funestus* from *An. gambiae*), given a 10% sugar solution and kept for 3–5 days until gravid. To obtain eggs from the gravid females, the forced egg-laying technique was used^49^. Harvested eggs were shipped to the Centre for Research in Infectious Diseases (CRID, Cameroon) for the rearing of progeny. Carcasses of the field mosquitoes (F0) were separated into oviposited and non-oviposited and stored in silica gel in Eppendorf tubes for DNA extraction.

### Mosquito rearing

All mosquitoes were reared under standard insectary conditions at a temperature of between 24 and 28 °C with 65–85% relative humidity and under a 12:12 photoperiod of natural light. Mosquito larvae were reared in larval trays and fed on Tetramine ad libitum. Larval water was changed every three days until pupation. Emerged adults were kept in Bugdom cages while being given 10% sugar solution for 3–5 days before bioassays.

### DNA extraction and molecular genotyping

For F0 mosquitoes, except for the October 2021 collection, the head and thorax were used for DNA extraction while for the F1s, whole mosquitoes were used. For all DNA extractions, the LIVAK protocol^50^ was used followed by storing the DNA samples in a − 20 °C freezer. To evaluate the vector bionomics; DNA from F0 mosquitoes were used to assess the species composition and sporozoite infection rates as well as resistance marker frequencies in the population. The species composition of the *An. funestus* population was assessed using the *An. funestus* species cocktail PCR^51^ and sporozoite infection rates were assessed using real-time Taqman PCR^52^. Sporozoite infection rates were assessed in the 524 mosquitoes that were identified by species in both oviposited and non-oviposited mosquitoes.

For the resistance makers, we sampled 30–40 mosquitoes from the 524 mosquitoes to assess the frequency of resistance makers in the population. We genotyped eight different validated markers; *Cyp6P9a*, *Cyp6P9b*, *L119F-Gste2*, *N485I-Ace1*, *A296S-Rdl*, *4.3Kb-SV*, *6.5Kb-SV* and *G454A-Cyp9k1*. For the *Cyp6P9a*, *Cyp6P9b* and *L119F-Gste2* markers, we designed new methods using the Locked Nucleic Assay (LNA)-based qPCR technique^53,54^ from Integrated DNA Technologies (IDT), Inc. to allow high throughput genotyping. The LNA-based protocols for these three markers are described in detail in the Supplementary section (Supplementary file 1). The remaining markers were genotyped using protocols described previously*; N485I-Ace1*^43^, *A296S-Rdl*^44^, *4.3Kb-SV*^38^, *6.5Kb-SV*^37^ and *G454A-Cyp9k1*^36^.

### Insecticide susceptibility tests

We conducted a series of bioassays to fully characterise the resistance of *An. funestus* populations at all the time points except for November 2023 due to low number of mosquitoes caused by insectary rearing conditions. From the emerged adult F1s, 3- to 5-day-old female mosquitoes were used for WHO tube assays, WHO cone assays, Bottle assays and Tunnel Tests using standardised protocols^55,56^. We examined the temporal patterns in insecticide resistance against standard diagnostic concentrations, including intensity assays and new bednets. Table 1 summarises the different bioassay tests we conducted using several insecticides and LLINs.

**Table 1:** List of insecticides or bednets used to characterise phenotypic resistance in *An. funestus* mosquitoes.

Exposure/insecticide	Class of insecticide	Concentration tested	Type of test
Permethrin	Pyrethroid, Type I	0.75% (1×), 3.75% (5×), 7.5% (10×)	WHO tube assay
Alpha-cypermethrin	Pyrethroid, Type II	0.05% (1×), 0.25% (5×), 0.5% (10×)	WHO tube assay
Deltamethrin	Pyrethroid, Type II	0.05% (1×), 0.25% (5×). 0.5% (10×)	WHO tube assay
DDT	Organochlorine	4% (1×)	WHO tube assay
Pirimiphos-methyl	Organophosphate	0.25% (1×)	WHO tube assay
Fenitrothion	Organophosphate	1% (1×)	WHO tune assay
Bendiocarb	Carbamate	0.1% (1×)	WHO tube assay
Piperonyl butoxide (PBO)	Synergist for P450s	4%	WHO tube assay
Diethyl Maleate (DEM)	Synergist for GSTs	4%	WHO tube assay
S,S,S-tributyl phosphorotrithioate (DEF)	Synergist for COEs	4%	WHO tube assay
Clothianidin	Neonicotinoid	4 µg (1×)	Bottle assay
Chlorfenapyr	Pyrrole	100 µg (1×)	Bottle assay
LLINs	–	–	WHO cone assay or Tunnel Test
PermaNet 2.0	Deltamethrin	120 mg/m^2^ of Deltamethrin	WHO cone assay
Permanet 3.0	Deltamethrin + PBO	84 mg/ m^2^ of deltamethrin on the sides and 120 mg/m^2^ on the top + 800 mg/m^2^ PBO	WHO cone assay
Olsyet 2.0	Permethrin	1000 mg/m^2^ of permethrin	WHO cone assay
Olyset Plus	Permethrin + PBO	1000 mg/m^2^ of permethrin + 1% PBO	WHO cone assay
Interceptor	Alpha-cypermethrin	200 mg/m^2^ of alpha-cypermethrin	WHO cone assay
Interceptor G2	Alpha-cypermethrin + chlorfenapyr	200 mg/m^2^ chlorfenapyr and 100 mg/m^2^ alpha-cypermethrin	Tunnel test
Royal Guard	Alpha-cypermethrin + Pyriproxyfen	208 mg/m^2^ of alpha-cypermethrin + 208 mg/m^2^ of Pyriproxyfen	WHO cone assay
Duranet	Alpha-cypermethrin	261 mg/m^2^ of alpha-cypermethrin	WHO cone assay

### Assessment of the transcriptomic profile of key genes

To temporally understand the mechanisms driving resistance escalation, we extracted RNA from alive mosquitoes after exposure to 1×, 5× and 10× alpha-cypermethrin and 1× permethrin while using unexposed 2021 mosquitoes and FANG lab strain as a field and susceptible control respectively. Relative to FANG, we examined the gene expression profile of six key genes that have previously been associated with pyrethroid resistance. We looked at the expression patterns of CYP9K1 (Vectorbase designation: AFUN007549), CYP6P9A (AFUN015792), CYP6P9b (AFUN015889), CYP6P5 (AFUN015888), CYP6M7 (AFUN007663) and GSTe2 (AFUN015809), genes that have been largely implicated in pyrethroid resistance in Uganda^32,57,58^. Briefly, in each group, total RNA was extracted from three batches of ten mosquitoes using the PicoPure® RNA Isolation Kit, (ThermoFisher Scientific, United Kingdom), followed by cDNA synthesis by reverse transcription using the SuperScript® III First-Strand Synthesis System, ThermoFisher (United Kingdom) and then finally quantitative polymerase chain reaction (qPCR) as previously described^59^. Gene expression was assessed and compared between the different groups by using the 2^−ΔΔCT^ method^60^ to calculate the relative expression and fold change after normalisation using the housekeeping genes ribosomal protein S7 (RPS7) (AFUN007153-RA) and Actin (AFUN006819-RA) as used previously^20,35^.

### Data analysis

Field and laboratory data were entered into an Excel sheet and analysed using R software (ver. 1.4.1106) packages to plot counts, proportions and frequencies. Gene expression data was plotted using R and Analysis of variance (ANOVA) online was used to compare the statistical difference in mean fold change expression levels between groups.

## Results

### Mosquito abundance and species composition

**Fig. 2:**
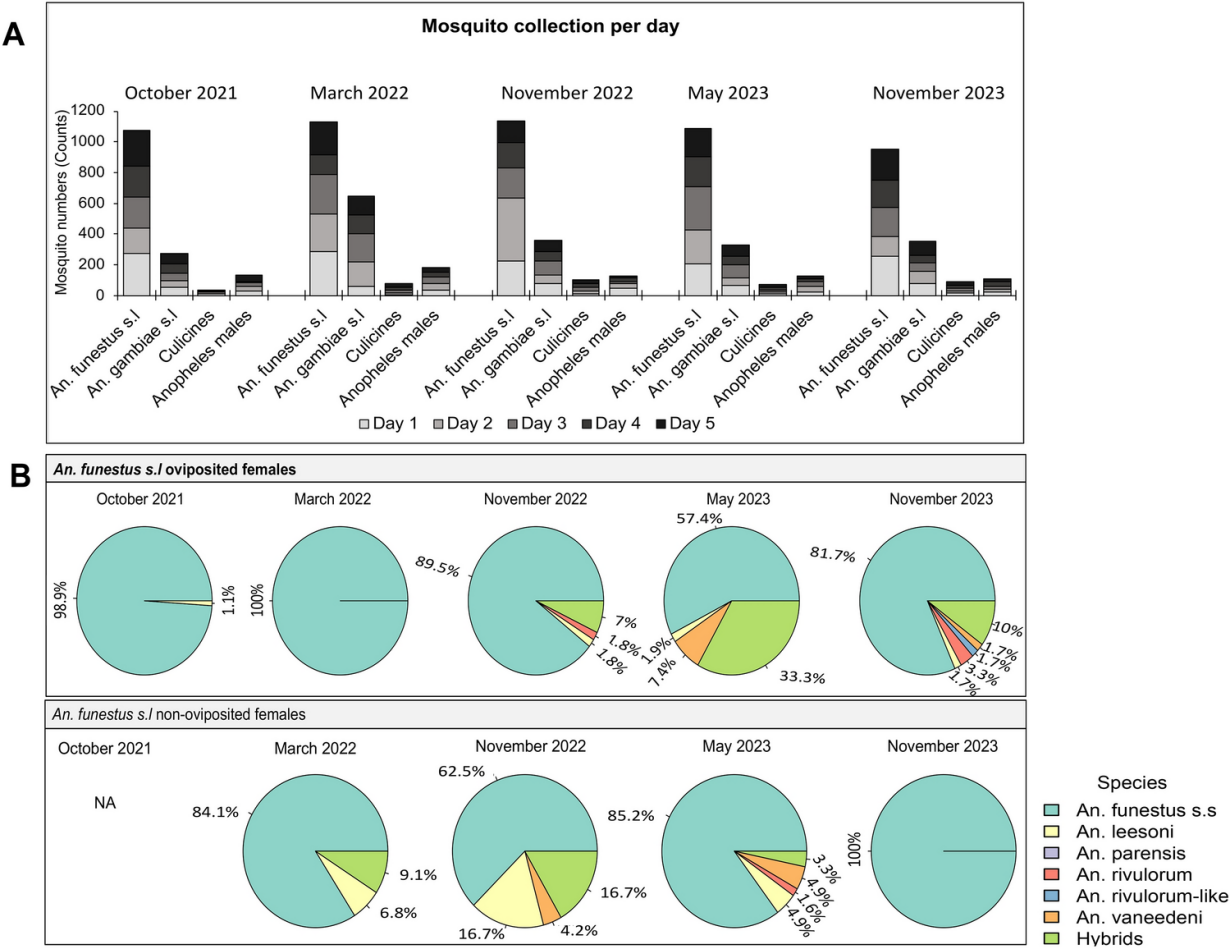
Abundance and composition of mosquito vectors in Mayuge. Total number of mosquito vectors collected over a five-day period for each time point (**A**) and *An. funestus s.l* species composition in ovipoisted and non-oviposited females over five time points from 2021 to 2023 (**B**).

The dominant malaria vector was *An. funestus* consistently from 2021, 2022 and 2023 with approximately more than 1000 individuals representing 55–71% of the collected mosquitoes (Figure 2A). From the collections, we sampled 40–60 field female mosquitoes (F0) from those that oviposited and did not oviposit to evaluate the species composition giving a total of 572 samples. The results from samples that amplified (n = 524) revealed a variation in species composition temporally (Fig. 2B). *An. funestus s.s* was still the most abundant species throughout the time periods (80–100% abundance) with significant relative reduction only in May 2023 in oviposited females (57.41%) and November 2022 in non-oviposited females (62.5%). We also detected other secondary species and these included; *An. leesoni, An. parensis, An. rivulorum, An. rivulorum-like* and *An. vaneedeni* at a relatively smaller proportions in oviposited compared to non-oviposited females. Interestingly, there was a high proportion of hybrids detected in the populations of *An. funestus* from Mayuge. However, these hybrids were not detected at all time points and the highest abundance was observed in May 2023 (33.3%) in oviposited females. Hybridisation species structure was very heterogenous although mostly comprised of *An. funestus s.s* and other secondary species (Supplementary file 1).

### Plasmodium sporozoite infection rates

**Fig. 3:**
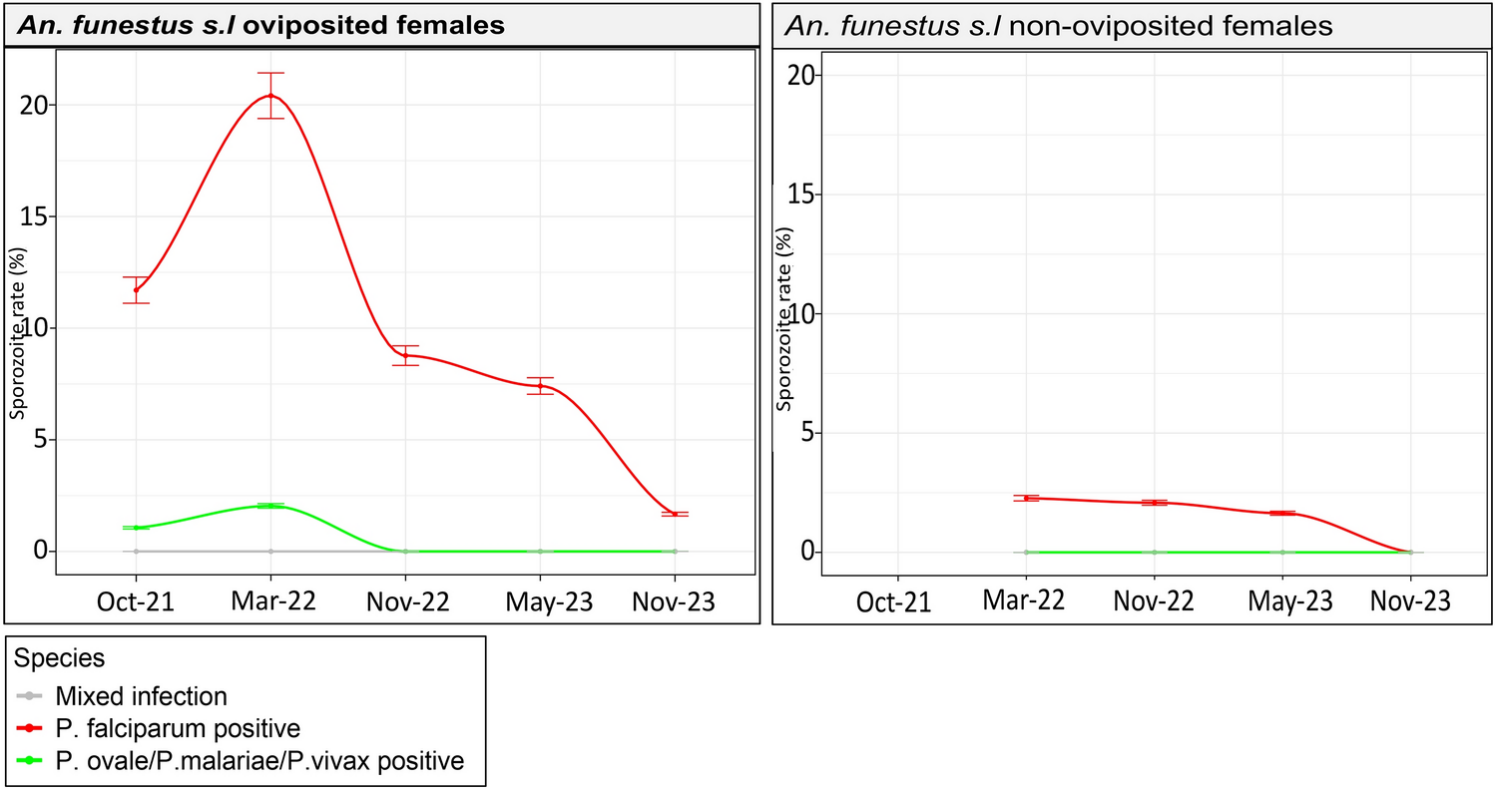
Sporozoite infection rates in field collected (F0) *An. funestus* mosquitoes. *Plasmodium* sporozoite infection rates in oviposited and non-oviposited wild *An. funestus* populations over five time points from 2021 to 2023. Error bars indicate a 5% error of the mean.

Sporozoite infections were predominantly *P. falciparum* (94.5%) and no mixed infections were detected in the samples (Fig. 3). In October 2021, sporozoite rates were 11.70% and 1.06% for *P. falciparum* and other *Plasmodium* species respectively in oviposited females. In March 2022, a peak infection rate was observed where 20.41% of the infections were *P. falciparum* in oviposited females while only 2.27% were observed in non-oviposited females. The percentage of other *Plasmodium* species was 2.04% in oviposited females and 0% in non-oviposited females. In November 2022, only sporozoite infection rates for *P. falciparum* were observed in oviposited females at 8.77%, while 2.08% was observed in non-oviposited females. The mosquito infection rates by *P. falciparum* in oviposited females dropped drastically from 7.41% in May 2023 to 1.67% in November 2023 and 1.64% and 0% in non-oviposited females in the same periods with no further observations of other *Plasmodium* species. Overall, oviposited females significantly (P < 0.001) carried the highest burden of sporozoite infection (91.7%) compared to non-oviposited females (8.3%). Within the parasite species, *An. funestus s.s* significantly (P < 0.001) carried the highest burden of sporozoites (87.2%) compared to other species (12.8%).

### Insecticide resistance profile

**Fig. 4:**
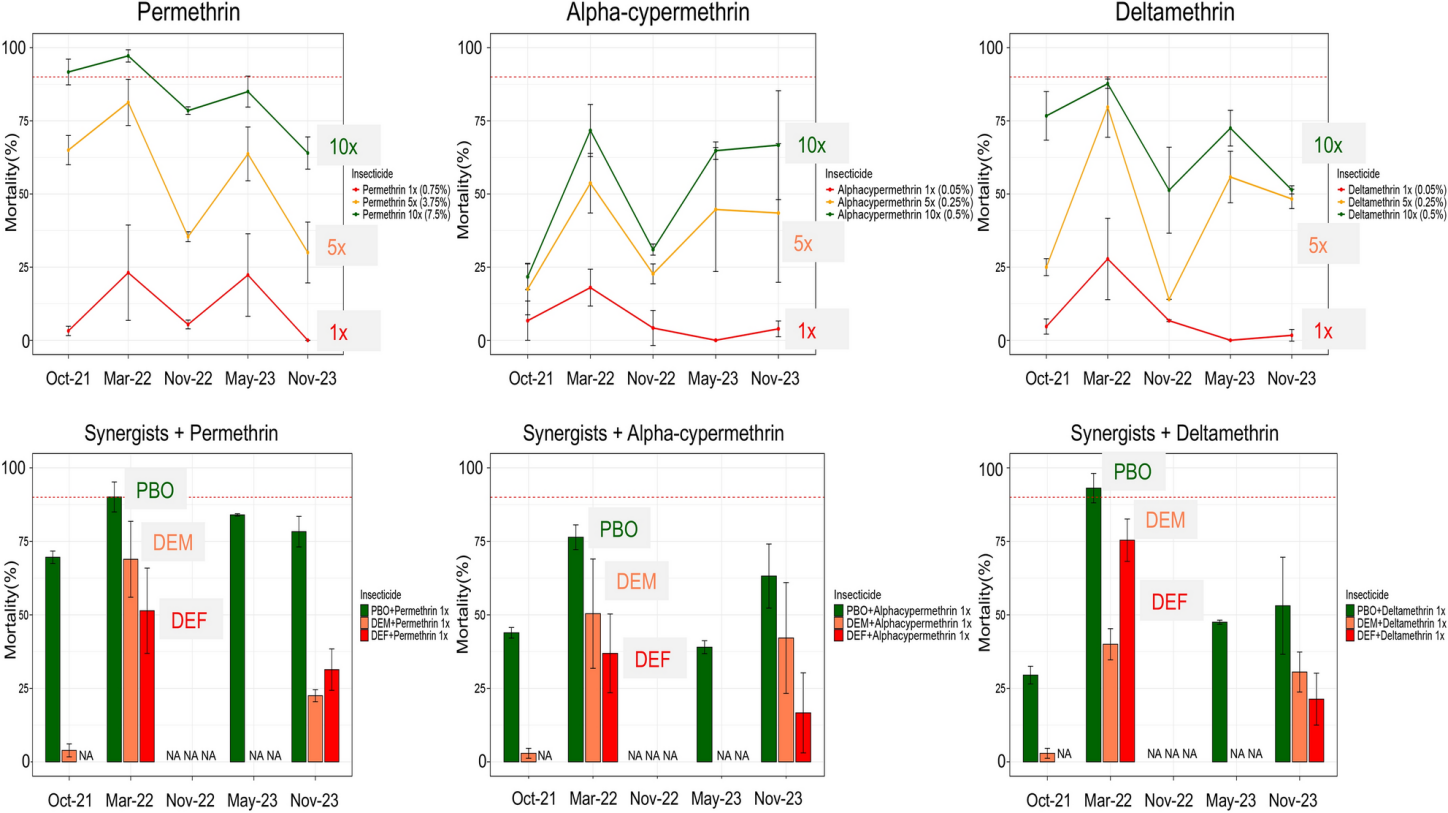
Mortality rates from F1 bioassays against pyrethroids. Point mortality rates of *An. Funestus* mosquitoes exposed to pyrethroids-only and pyrethroids + synergists. Error bars in the line graph and bar chart indicate confidence intervals calculated by SEM and NA indicate tests that were not performed. The red dotted horizontal line is 90% mortality cut-off, below which is confirmed resistance.

Insecticide resistance and intensity were more pronounced in type II pyrethroids (deltamethrin and alpha-cypermethrin) compared to type I pyrethroid (permethrin) (Fig. 4). Increasing dosage greatly increased mortality with permethrin (average mortality; 1× = 10.8%, 5× = 55.1% and 10× = 83.3%) but not so much with alpha-cypermethrin (average mortality; 1× = 6.6%, 5× = 36.4% and 10× = 51.2%) and deltamethrin (average mortality; 1× = 8.2%, 5× = 44.6% and 10× = 67.9%) (Figure 4). Similarly, pre-exposure to synergists notably increased the mortality with permethrin with PBO (average mortality = 80.5%) compared to DEM (average mortality = 31.8%) and DEF (average mortality = 41.4%) although full restoration of susceptibility was not achieved. However, a lower increase in mortality especially with PBO was observed with alpha-cypermethrin (average mortality; PBO = 55.6%, DEM = 31.8% and DEF = 26.8%) and deltamethrin (average mortality; PBO = 55.5%, DEM = 24.5% and DEF = 48.4%). The temporal pattern in pyrethroid resistance was variable between different months but almost alternating around the same point. However, there was a noticeable trend in decrease of mortality to permethrin at higher doses (Fig. 4). Similar patterns were observed with the knockdown where rates were almost reflective of the observed mortality (Supplementary Figure 2).

**Fig. 5:**
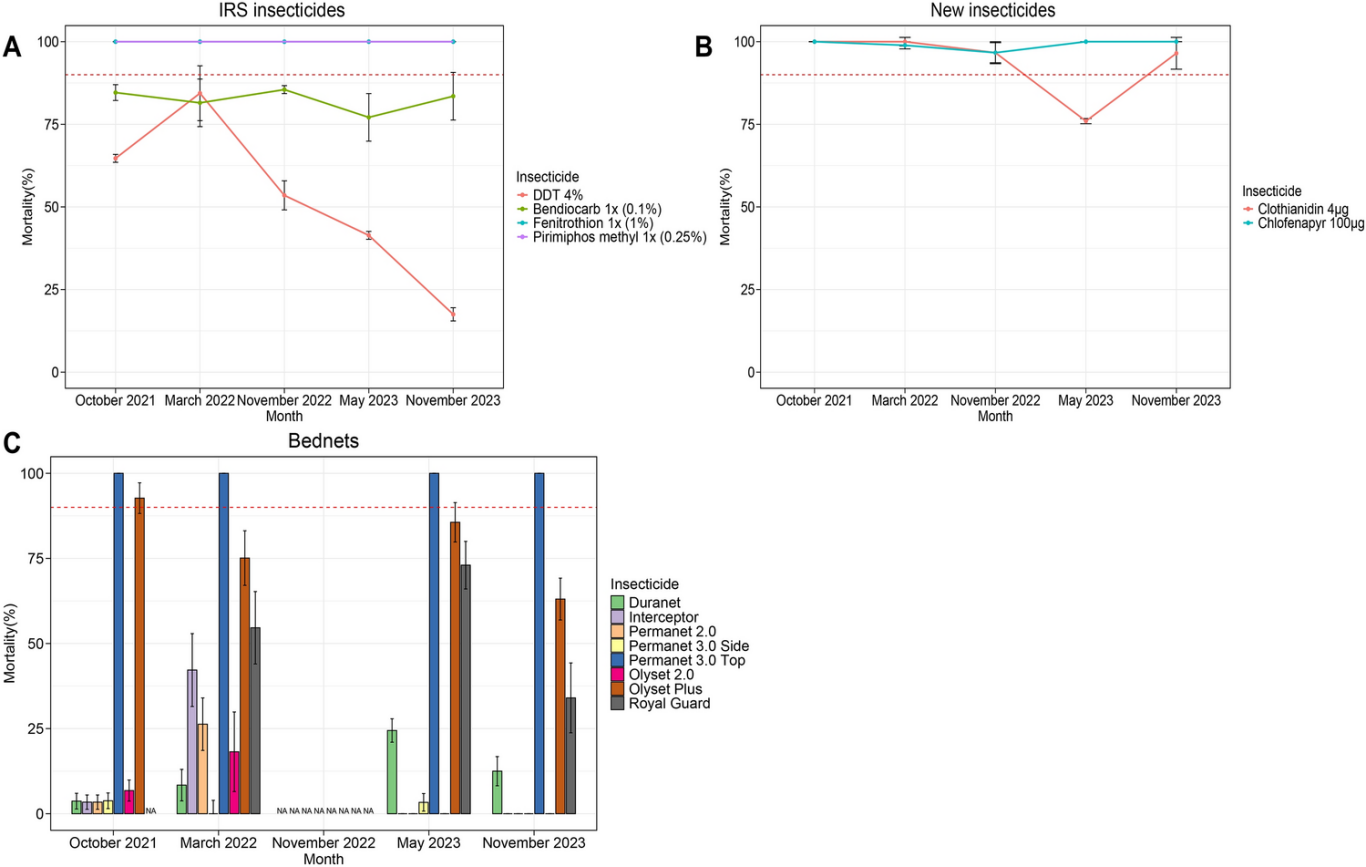
Mortality rates from F1 bioassays against insecticides used in IRS and bednets. Point mortality rates for insecticides DDT, Bendiocarb, Pirimiphos-methyl (**A**); clothianidin and chlorfenapyr (**B**) and LLINs (**C**). Error bars in both line and bar graph indicate confidence intervals calculated by SEM. NA indicate tests that were not performed. The dotted red horizontal line is 90% mortality cut-off, below which is confirmed resistance.

Insecticide resistance patterns against other insecticides were more consistent except for DDT insecticide. Mortality rates for DDT drastically decreased from 64.7% in 2021 to 17.5% in 2023 (Fig. 5A). This pattern was also observed with the knockdown rates (Supplementary Figure 3A). Mortality rates for Bendiocarb were consistent over the years with an average of 82.4% but the knockdown rate was very high (up to 100% in most months) (Supplementary Figure 3A). By contrast, mosquitoes were fully susceptible to organophosphates (pirimiphos-methyl and fenitrothion). Similarly, although there was largely no resistance to neonicotinoids and pyrroles, there were signals of reduced susceptibility as observed by decreased mortality for clothianidin in May 2023 (Fig. 5B) and relatively low knockdown rates throughout the years (Supplementary Figure 3B).

When mosquitoes were exposed to bednets, dual-active ingredient LLINs produced higher mortality rates. Permanet 3.0 Top produced a very high kill rate (average mortality = 100%), followed by Olyset Plus (average mortality = 79.1%) (Figure 5C). However, unlike Permanet 3.0 Top, knockdown rates (average = 93.4%) (Supplementary Figure 5) for Olyset Plus were higher than the mortality rates. Although these rates did not vary considerably between the months of collection, there was a noticeable trend in the decrease of efficacy of Olyset Plus (Fig. 5C). Among the dual active ingredient nets, the chlorfenapyr-based Interceptor G2, produced a mortality rate of 70.1% in November 2022 (Supplementary Figure 4) while Royal Guard had an average mortality rate of 53.9% with slightly reduced efficacy in November 2023. Single active ingredient LLINs had very poor efficacy throughout the months of testing with higher mortalities observed only in March 2022. Duranet (average mortality = 12.2%) and Interceptor (average mortality = 11.4%). Knockdown was seemingly higher than mortality for most of the single-ingredient LLINs (Supplementary Figure 5).

### Allele frequency of resistance markers

**Fig. 6:**
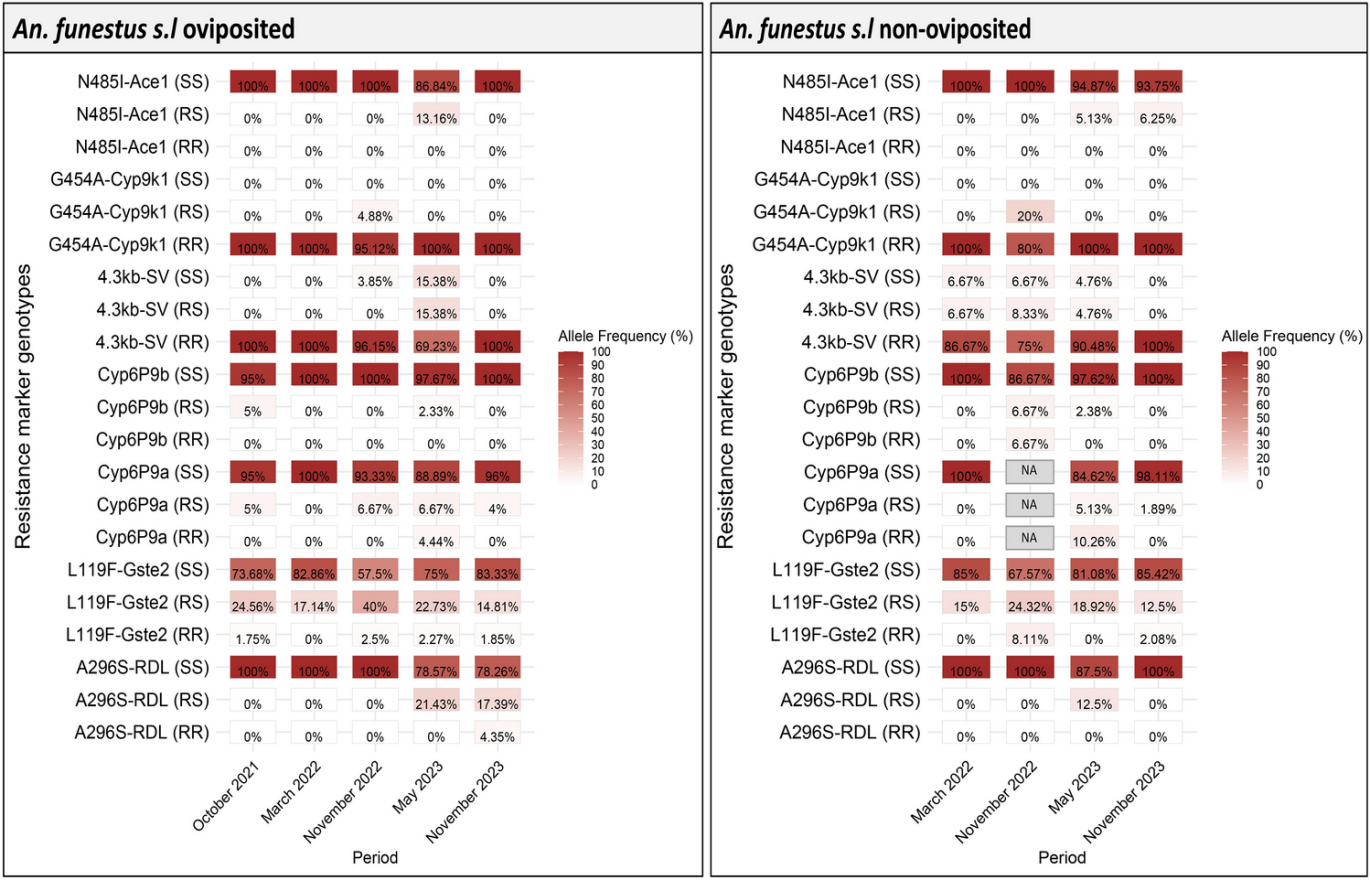
Insecticide resistance marker prevalence across five time points from 2021 to 2023. Point prevalence of major insecticide resistance markers in *An. funestus*. The different genotypes are shown in brackets and represented as; SS for homozygous susceptible/wild type, RS for heterozygous and RR for homozygous resistant/mutant.

There was no drastic change in the frequency of resistance markers over the five time points and the homozygous resistant genotypes (RR) of most markers were either very low, fixed or absent in the population. Most of the notable variations were seen in November 2022 and May 2023 (Figure 6). We did not detect the *6.5kb-SV* marker in any of the samples using the current method.

The frequency of the RR genotype of the *L119F-Gste2* marker was at very low frequency with no major changes between 2021 and 2023 though there was an increase in RS (heterozygous) genotype in oviposited females (40%) in November 2022 which was also marked with a slight increase of RR genotype in non-oviposited females (8.11%). Similar observations were seen with *Cyp6P9a* and *Cyp6P9b* markers where the frequency of RR and RS genotypes were very low with some few variations in mosquitoes that oviposited but slightly higher frequencies in non-oviposited females which will require further investigation.

The *G454A-Cyp9k1* and *4.3kb-SV* markers were generally fixed in the population but some variations in frequency were observed. In May 2023 among the oviposited females, the frequency for RR genotype for *4.3kb-SV* was only ~70% with the detection of RS and SS (homozygous susceptible) genotypes in the population but the *G454A-Cyp9k1* marker remained fixed throughout. In non-oviposited females, all genotypes for *4.3kb-SV* were detected at all time points except November 2023 and this included a detection of only 75% of RR genotype in November 2022. The RR genotype of *G454A-Cyp9k1* was fixed throughput except in November 2022 where the frequency was only at 80%.

Uniquely, we have detected the presence of resistant alleles for *A296S-Rdl* and *N485I-Ace1* in *An. funestus* populations but at very low frequencies. These makers were detected only in May 2023 and November 2023. For the *N485I-Ace1,* only the RS genotype was detected in the population (13.6% in oviposited and 5.13% in non-oviposited) while for the *A296S-Rdl,* both RS and RR genotypes were detected (RS = 21.43%, RR = 4.35% in oviposited) with the latter being detected only in November 2023. These results mark the first-ever report of detection of the resistant alleles for *A296S-Rdl* and *N485I-Ace1* in East Africa.

### Transcriptomic profile of key genes

**Fig. 7:**
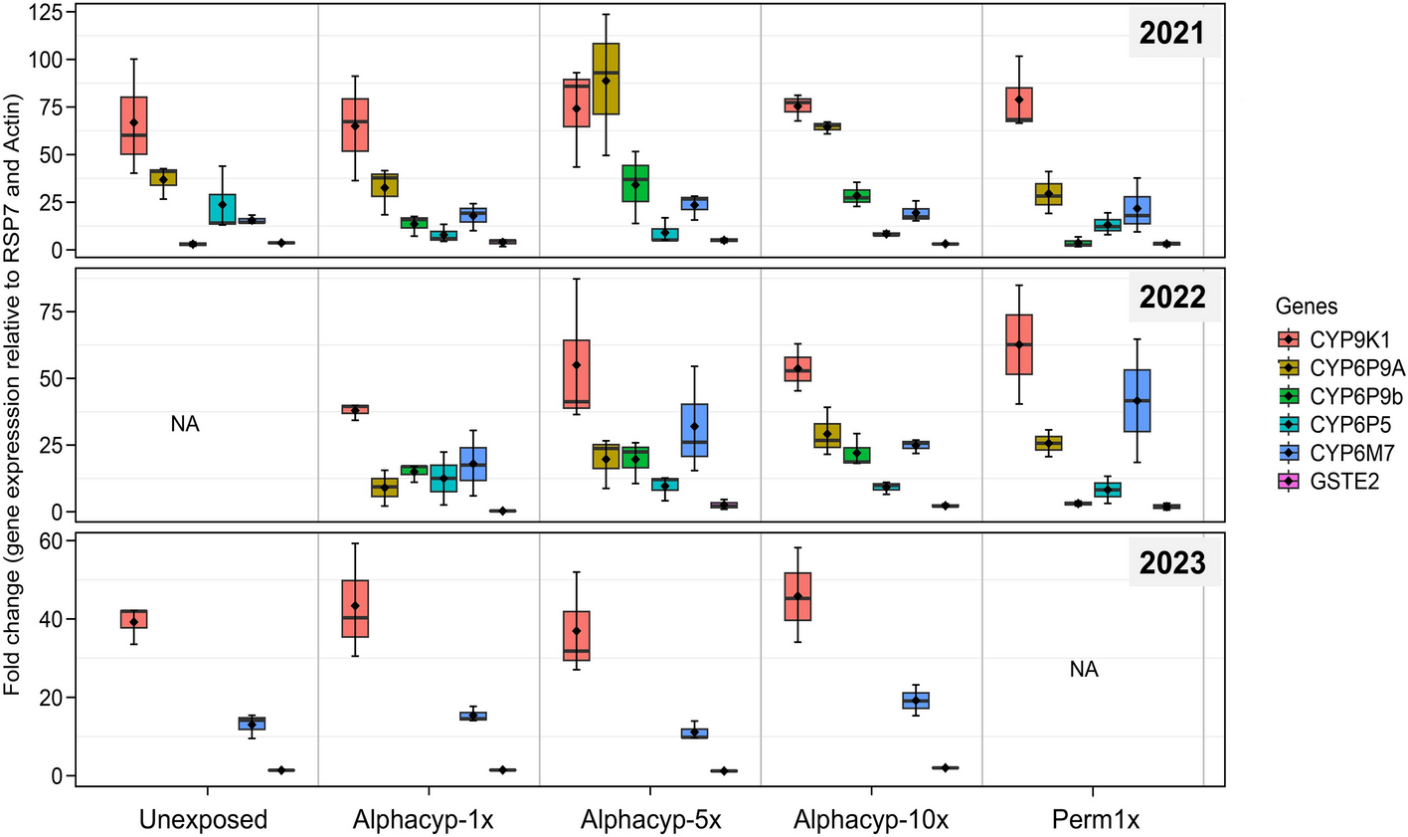
Fold change analysis of the expression of key genes across three years. Box plot shows the mean fold change expression of six key genes from three biological replicates. Error bars represent SEM. Abbreviated labels on the x-axis are; “Alphacyp” = Alpha-cypermethrin and “Perm” = Permethrin with the corresponding insecticide intensity of 1×, 5× and 10× as explained in Table 1. NA means the group was no assessed. Genes CYP6P9A, CYP6P9b and CYP6P5 were not analysed in 2023.

Fold change (FC) analysis revealed the overexpression of CYP9K1 and the two duplicated genes (CYP6P9A and CYP6P9b) as the key drivers of resistance although only CYP9K1 had consistent results (Fig. 7). The fold change (FC) analysis showed that CYP9K1 gene was consistently overexpressed across the different exposure groups and time periods. For example, in mosquitoes exposed to permethrin, the FC for CYP9K1 gene was 78.7 ± 11.4 in 2021 and 62.7 ± 18.2 in 2022. Similarly, for alpha-cypermethrin, the FC ranged from 65.0 ± 15.9 in 2021 to 45.8 ± 12.1 in 2023, with no significant differences between exposure groups or doses (One-way ANOVA; df = 12, F = 0.6, P = 0.674). However, there was a significant difference in gene expression temporally, with higher levels observed in 2021 than in 2022 (One-way ANOVA; df = 12, F = 21.6, P = 0.00024). The CYP6M7 gene also showed consistent expression but at lower levels, with FC values around 17.9 ± 4.2 in 2021 and 15.4 ± 1.1 in 2023 for alpha-cypermethrin. Similarly, CYP6P9A and CYP6P9b were highly upregulated in 2021 (e.g., CYP6P9A FC = 32.6 ± 12.4 for alpha-cypermethrin 1× in 2021 and CYP6P9b FC in alpha-cypermethrin 1× was 13.6 ± 3.2 in 2021. Both genes showed higher expression with type II pyrethroids and increased doses, such as CYP6P9A FC reaching 88.7 ± 21.5 for alpha-cypermethrin 5× in 2021. The CYP6P5 gene followed a similar pattern to CYP6P9b, with FC dropping from 23.7 ± 10.1 in 2021 in unexposed mosquitoes. Meanwhile, GSTE2 had very low FC (< 3 ± 0.5) and did not show significant changes (P = 0.2611) in expression across the different exposure groups and time points.

## Discussion

Insecticide resistance is the biggest threat to the continued and sustainable use of insecticide-based mosquito control tools. Worrying levels of intense insecticide resistance have been widely reported further shrinking the choices available for effective vector tools. Practically, insecticide resistance is very dynamic and vector populations will respond to the applied selection pressure which consequently alters the population structure. Our study monitored insecticide resistance-associated dynamics for an extended time in *An. funestus* populations from eastern Uganda during a period when different vector control tools were being deployed by the National Malaria Control Program (NMCP).

### Temporal dynamics in the bionomics of *An. funestus* species in Uganda reveal population admixture and changes in sporozoite infection rates

Since 2010, malaria transmission has been principally driven by *An. funestus* especially in East and Southern Africa, largely attributable to the scale-up of LLINs over the last 15 years^46^. It is argued that *An. funestus* was likely less affected by insecticides used for treatment of bednets notably pyrethroids due the ability to rapidly develop strong resistance to Insecticide treated net (ITNs). This resistance, combined with its preference for feeding on humans, long lifespan, tendency to take multiple blood meals, and behaviours such as breeding throughout the year^61^, resting indoors and biting in the early morning^62,63^, enhanced its survival and chances to be highly and efficiently infected with *Plasmodium* parasites hence driving transmission of malaria. Furthermore, when bednets are in use, *An. funestus* has been observed to be capable of penetrating through the net to blood feed^64^. Consistent with this, the population of malaria vectors we surveyed were predominantly *An. funestus s.l* which is also consistent with the study conducted in 2020^9^. However, the temporal investigation revealed that the population of *An. funestus* may not be stable with only one species i.e. *An. funestus s.s* dominating. We observed a reasonable presence of other sibling species and notably, high but unstable hybridization rates signalling population admixture among *An. funestus s.l*. Similar observations were observed in the Chikwawa region of Malawi in 2014 and 2021 which also happened to coincide with intense and multiple insecticide resistance in Malawi^11,65^. These changes in *An. funestus* population structure seems to reflect a response to the different vector control tools that were rolled out by NMCP in Uganda. In 2019-2020, when Actellic (pirimiphos-methyl) was the principle insecticide used for IRS in eastern Uganda^2^, the population was mainly *An. funestus s.s* in Mayuge^20^. Uganda switched to a clothianidin-based formulation in 2020–2022 for IRS, which was marked by a dramatic malaria resurgence mainly driven by clothianidin-tolerant *An. funestus*^2^. During the clothianidin IRS period, *An. funestus* thrived, leading to an apparent increase in their abundance^2^ hence providing a suitable pool and condition for population admixture. Hybridization rates in *An. funestus* population occur more often than in *An. gambiae* population and are corridors of introgression of genetic elements such as resistance genes into the main vector population. However, when Actellic was re-introduced in March 2023, by November 2023 the population was almost similar to that observed in 2020 with the majority being *An. funestus s.s*. The impact of the different IRS periods in Uganda is accurately aligned with this study and was probably a big factor that influenced mosquito bionomics in the area. For instance, sporozoite rates were higher than observed in February 2020^20^ with a peak in March 2022 which happened to coincide with the peak of the malaria resurgence in eastern Uganda. Shortly after the re-introduction of Actellic, we observed a reduction in sporozoite rates in May 2023 with a further drastic decrease by November 2023 to a lower rate similar to that observed in Tororo and Busia around the same period^2^.

Interestingly, we observed higher sporozoite rates in oviposited mosquitoes compared to non-oviposited mosquitoes. It is unclear why this was the case given that the opposite was observed in February 2020 by Tchouakui et al. A probable explanation is likely linked to the effective IRS campaigns and distribution of LLINs conducted between 2018 and 2020 on the fitness of the mosquito populations. The naturally selected clothianidin-tolerant *An. funestus* populations during the malaria resurgence period must have been fitter since this was an entirely new formulation that had never been used in Uganda. Studies have shown that increased insecticide selection pressure on mosquitoes can lead to higher sporozoite infection rates^66^ and resistant mosquitoes generally tend to carry a higher parasite burden^67–71^ which increases their egg oviposition rates^70^ This interplay between mosquito parasite infection and oviposition is very complex to understand. Parasites such as *Plasmodium* are known to manipulate their hosts to sometimes bring forth oviposition to allow another cycle of blood-feeding which potentially increases the chance of being transmitted through the next blood meal^72^.

### Genetic mechanisms and drivers of insecticide resistance in *An. funestus* from Uganda and their temporal changes between 2021 and 2023

Despite the visible changes in the mosquito population and bionomics, there was no marked effect on the allele frequency of most of the resistance markers except the *A296S* and *N485I* markers. Additionally, the allele frequency of the markers cannot explain the observed phenotypic resistance or parasite infection. Pyrethroid resistance intensification was first reported in Uganda in 2020^20^. Since 2020, not much has changed but there are indications of further decreasing efficacy of permethrin at 10x diagnostic dose which might also be further affecting the performance of Olyset Plus. Additionally, the escalation is worse with type II pyrethroids, possibly alluding to the different resistance mechanisms involved. Indeed, PBO synergistic assays revealed higher mortality rates with permethrin exposure than alpha-cypermethrin and deltamethrin.

In May 2023, we detected both mutant alleles for *Rdl-A296S* and *Ace1*-*N485I* markers for the first time. Only *Rdl-A296S* had a temporal increase in the homozygous mutant allele in November 2023 while the *Ace1*-*N485I* marker was only detected in heterozygous form and seemed unstable. The detection of these two markers possibly indicates an introgression that might have occurred during the resurgence or a selection pressure or both with the period likely earlier than May 2023. The *Rdl-A296S* marker has been detected before in Uganda in 2009^44^ but was not detectable by 2020^20^. The *Rdl-A296S* is a West African marker associated with dieldrin resistance hence it’s not associated with any of the insecticides that are currently used in Uganda. Contrastingly, it is the first time the *Ace1*-*N485I* marker has been detected in Uganda. The marker was first detected only in Southern Africa^43^ where it conferred cross-resistance to pyrethroids and carbamates. Currently, the intense pyrethroid resistance and moderate carbamate resistance detected in Uganda cannot possibly be associated with the *Ace1*-*N485I* marker due to its very low frequency and because carbamate was discontinued for IRS in 2016. So, it is unlikely that there will be further selection of this marker unless there is an unknown pressure from agricultural pesticides or other sources which will also entirely depend on the fitness costs involved with harbouring the allele.

It appears that most of the mechanisms conferring pyrethroid resistance in Uganda exist largely within the *rp1* QTL including the 4.3kb transposon^31,32,38^ and within the CYP9K1 haplotype^32,36^. Indeed, gene expression analysis revealed stable upregulation of CYP9K1 and CYP6M7 in *An. funestus* population from eastern Uganda but it is unlikely they are involved in pyrethroid resistance escalation hence, other key mechanisms exist. This is further supported by evidence where the *G454A CYP9K1* marker and *4.3-Kb SV* are already fixed in Uganda hence, they can’t explain the resistance escalation observed although it is currently unclear how far these markers have spread within Uganda since their distribution can vary within the country, as seen in Cameroon and across geographical locations^36,73^. Mechanisms driving pyrethroid resistance in Uganda seem to be very complex to map especially in eastern Uganda largely because of the different selection pressures arising from the rotation of multiple malaria control interventions including both LLINs and IRS^2,15^ and agricultural pesticides. For instance, between 2011 and 2012, microarray-based transcriptomic analysis revealed that key genes expressed in *An. funestus* populations from Uganda included CYP9K1, CYP6M7 with significant variations in expression levels of CYP6M7 between northern and eastern regions^58^ but low relative expression of GSTE2, CYP6P9A and CYP6P9b genes^74^. In 2021-2022, we found similar results but contrastingly, high expression of CYP6P9A and CYP6P9b genes which was also similar to what was observed in 2020^20^. However, the high overexpression did not associate with the southern African *CYP6P9a/b* markers^34,35^ which were at very low frequencies, but rather is associated with the presence of the 4.3kb structural variants found only in East and Central Africa^38^.

### Impact of malaria control programs in Uganda on insecticide resistance in *An. funestus*

Malaria control campaigns in Uganda since 2009 has been dominated by the distribution of bednets every three years with the key introduction of PBO nets in 2017 which were then complemented by other types of bednets like Pyriproxyfen-based nets in 2020 and Chlorfenapyr-based nets in 2023. Despite the recent reduction in the efficacy of especially PBO nets^12,18,75^, these LLINs were largely effective including in reducing vector abundance^15,76^. Indeed, cone assays revealed that PBO and chlorfenapyr nets offer a very high killing effect on highly resistant *An. funestus* populations although efficacy in the field is better measured by experimental hut trials^77,78^. Supplementary to LLINs, Uganda also conducts IRS campaigns in high transmission areas^79^ and since 2009, rotation was made between Actellic and Bendiocarb and then recently introducing Fludora Fusion and Sumishield in 2020–2022 before switching back to Actellic in 2023^2^. Actellic remains the only fully effective IRS formulation in Uganda as also witnessed by the full susceptibility of the multi-resistant *An. funestus* populations to pirimiphos-methyl in this study. However, there is resistance to Bendiocarb which might have occurred earlier or shortly before the formulation was discontinued in 2016. Carbamate resistance is now possibly being driven by cross-resistance with pyrethroids-associated genes as seen in Malawi^43^ with the selection pressure from bednets. Additionally, although *An. funestus* were largely susceptible to clothianidin, there were signals of resistance to these chemicals as witnessed in this study over time which is in line with the recent surge in malaria cases driven by *An. funestus* when clothianidin-based formulations were introduced^2^. The mechanisms that drove clothianidin tolerance/resistance were not further investigated. Although *An. funestus* populations remains largely susceptible to clothianidin^80^, resistance has been reported in *An. gambiae* from Cameroon^81^. A recent study has shown that cross-resistance can occur with pyrethroids with an association with the *L119F-GSTe2* marker^80^. However, the low frequency of the *L119F-GSTe2* marker and low expression of the GSTE2 gene observed in this study cannot explain the observed phenotypic resistance including against pyrethroids, DDT or bednets. Besides cross-resistance to clothianidin, the *L119F-GSTe2* marker is known to confer DDT resistance in West Africa^42^ with cross-resistance to pyrethroid further decreasing the efficacy of bednets^18^. Contrastingly, studies have shown that the *L119F-GSTe2* marker together with key CYPs such as *CYP6P9a/b* markers show a negative association with chlorfenapyr^82,83^. We did not detect any chlorfenapyr resistance which was also synonymously evidenced by the high killing effect of Interceptor G2, a chlorfenapyr-based bednet, in this study. One major reason for detecting no chlorfenapyr resistance could be that the key mechanisms which are mainly CYPs in *An. funestus* are rather promoting chlorfenapyr susceptibility rather than resistance as shown recently in Anopheles vectors^83^. It will therefore be interesting to see how the mosquito population structure and resistance patterns/mechanisms change after the 2023 rollout of approximately 330,000 chlorfenapyr-based bednets in Mayuge district and country-wide.

## Conclusion

The evolution of insecticide resistance is very dynamic, often rapid and can lead to mosquito population changes. Here, we see how complex insecticide resistance and its associated mechanisms is in *An. funestus* from Uganda which might principally be driven by overexpression of multiple metabolic genes with key genetic markers. The deployment of various insecticide-based tools in Uganda, primarily pyrethroid LLINs, does not seem to significantly affect the allele frequency of genetic markers at least in Mayuge district potentially due to the near fixation of major P450 resistance alleles. However, the IRS program seems to have a real-time impact on mosquito population structure and bionomics highlighting the importance of rotating interventions and implementing new insecticides. Upregulation of multiple key gene families such as CYP9K1, CYP6P9A/b, CYP6P5 and CYP6M7 seems to be the major strategy deployed by Ugandan *An. funestus* to counter pyrethroids and possibly other insecticides. This means that any adaptive selection of genetic variants such as SNPs and transposons will likely be linked to significant overexpression of major genes as seen with the *CYP9K1 G454A* single allele and *4.3-Kb* transposon in *An. funestus*^36,38^, and the *CYP6P4* triple mutant allele with *Zanzibar (ZE)* transposon in *An. gambiae*^84^ from Uganda. This selective advantage of such SNPs or transposons would lead to fixation within a short time and remain fixed as long as the same selection pressure is applied and there is minimal fitness cost attached to the genetic variant. It is very likely that within the key upregulated genes in *An. funestus* populations from Uganda, other key genetic variants exist and auxiliary temporal genomic monitoring should continue to identify these. Additionally, further studies to investigate mechanisms of resistance such as apparently seen with clothianidin and bendiocarb are recommended.

## Supplementary Information

Supplementary Information 1.

Supplementary Information 2.

## Data Availability

All datasets generated or analysed during this study are included in this published article and its supplementary information files.
