# Supplementary material for: Temporal evolution of insecticide resistance and bionomics in Anopheles funestus, a key malaria vector in Uganda
Source: Sci Rep. 2024 Dec 30;14:32027. doi: 10.1038/s41598-024-83689-6 (PMC11685729; doi:10.1038/s41598-024-83689-6)
Supplement: Supplementary file 1 — Supplementary Information 1. [file 41598_2024_83689_MOESM1_ESM.pdf]

Supplementary Figures

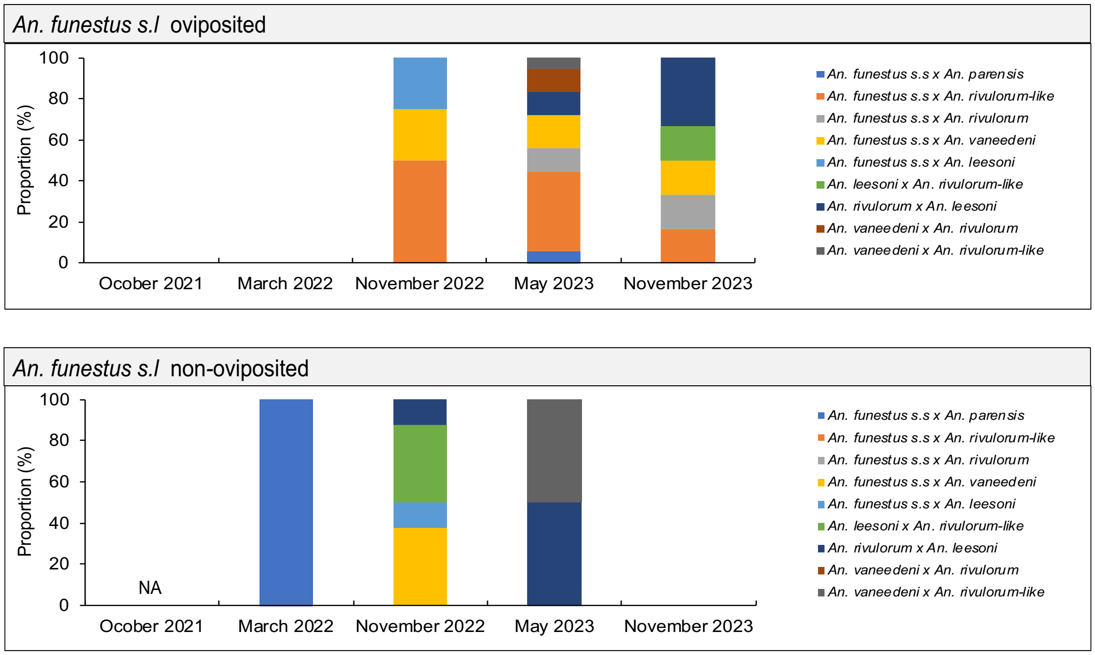

Supplementary Figure 1. Break down of the composition of hybrids detected in *An. funestus* populations in Mayuge over five time points from 2021 to 2023.

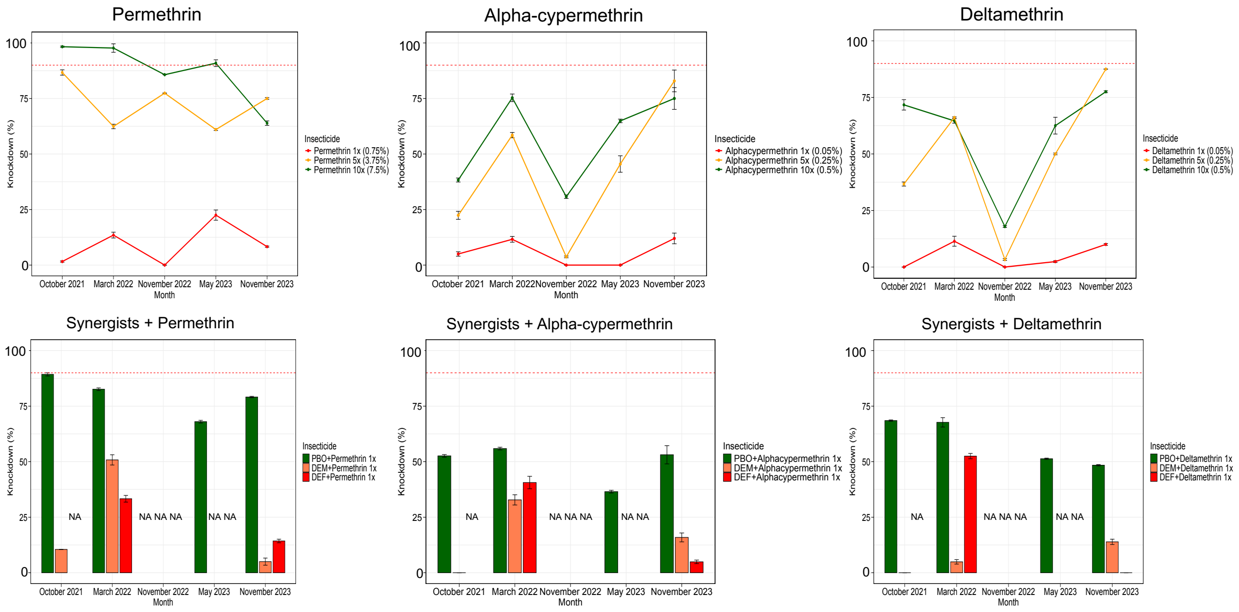

Supplementary Figure 2. Knockdown rates of mosquitoes exposed to pyrethroids-only and pyrethroids + synergists. Error bars indicate confidence intervals calculated by SEM. NA indicate tests that were not performed. The red horizontal line is 90% mortality cut-off, below which is confirmed resistance.

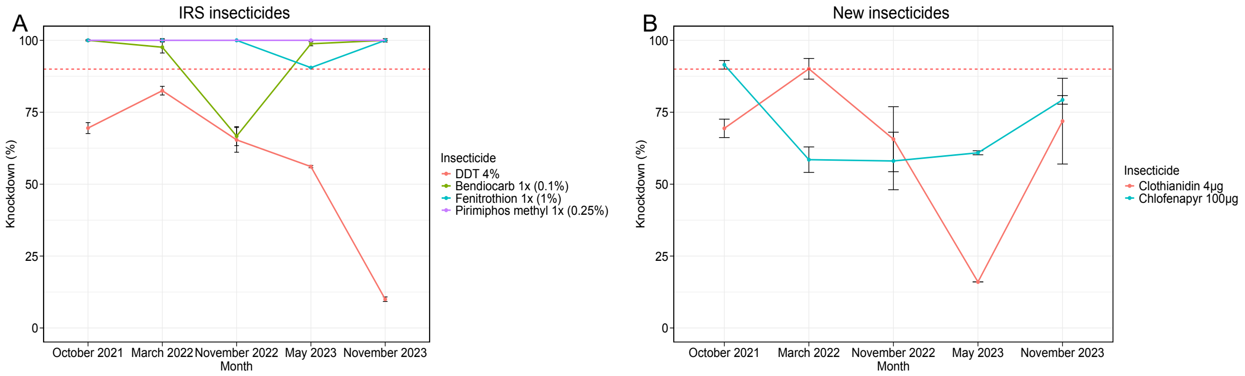

Supplementary Figure 3. Knockdown rates of mosquitoes exposed to IRS active ingredients including the new insecticides. A) represents knockdown rates for IRS insecticides (DDT, Bendiocarb, Pirimiphos-methyl) and B) represents the knockdown rates for newly prequalified insecticides (clothianidin and chlorfenapyr). Error bars indicate confidence intervals calculated by SEM. The red horizontal line is 90% mortality cut-off, below which is confirmed resistance.

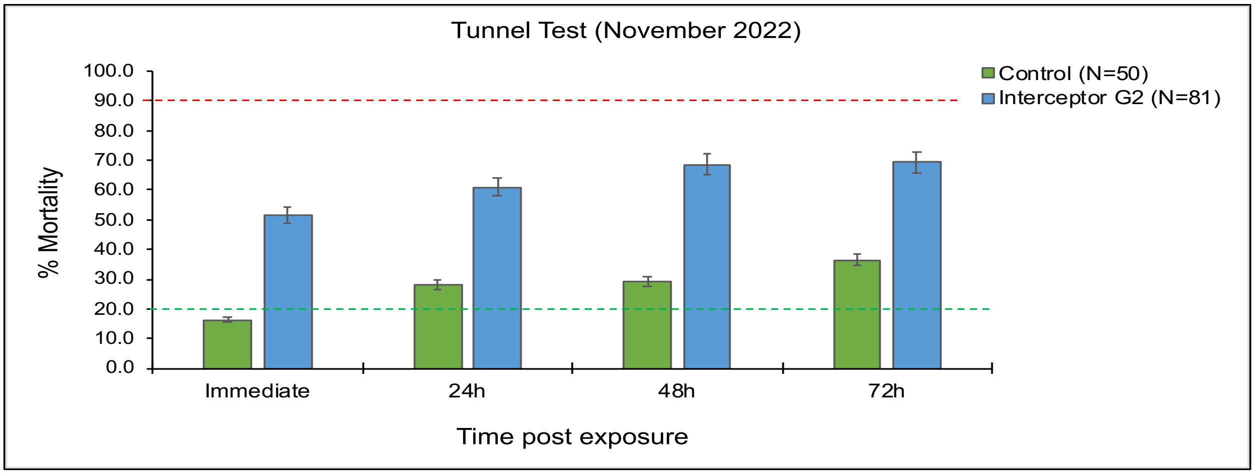

Supplementary Figure 4. Tunnel test to assess the mortality rate of Interceptor G2 conducted in November 2022. Error bars are 5% SEM. The red horizontal line is 90% mortality cut-off, below which is confirmed resistance. The green line is the maximum mortality acceptable for controls.

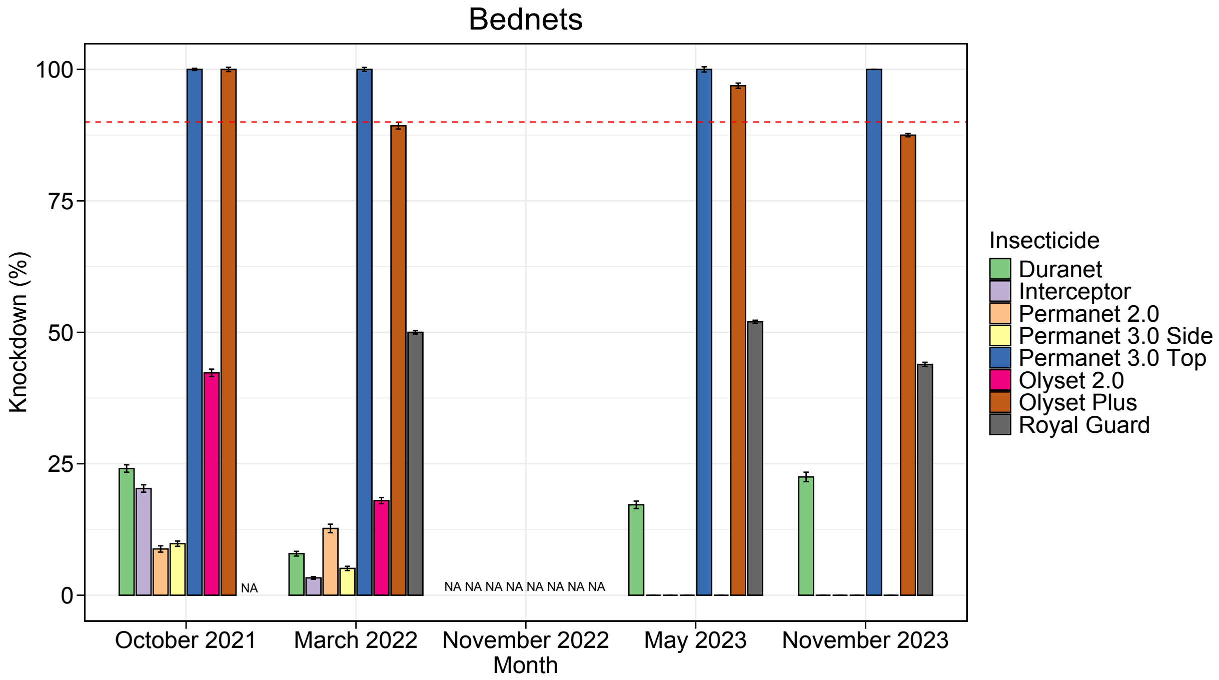

Supplementary Figure 5. Knockdown rates of mosquitoes exposed to LLINs. Error bars indicate confidence intervals calculated by SEM. NA indicate tests that were not performed. The red horizontal line is 90% mortality cut-off, below which is confirmed resistance.
