# Supplementary material for: Temporal evolution of insecticide resistance and bionomics in Anopheles funestus, a key malaria vector in Uganda
Source: Sci Rep. 2024 Dec 30;14:32027. doi: 10.1038/s41598-024-83689-6 (PMC11685729; doi:10.1038/s41598-024-83689-6)
Supplement: Supplementary file 2 — Supplementary Information 2. [file 41598_2024_83689_MOESM2_ESM.docx]

**Supplementary methods:**

1. **Protocol for genotyping the CYP6P9a marker in *An.funestus* using Locked Nucleic Acid (LNA) Assay**

Gene sequence:

TTTTTACAATGATAACCAATTTTTTATTTCTACAAGTAGATATCCTTAATTTTCAATAATCCGCTGTTATAACATTACGGGAAGATATGAAAAAATATAATGACTATTGACTGTGACTAAATGACTATAAGATGTTTACCTCAATTTGAACTACAAAATTGAGATTAATTTAATAAAATAACGAGACTGACATTTTAGTGTGTCTGTAACTGTAAATGATCCCTAACTATTAAAAGGCAATTACCTCCGTACGTTTCGGTGCACTTATGAACAAAGCTGTTGAAATCGTTTTAATGATAAAGTCAAGAAATTACATGTTTCAAGTCACCTGCACTTGAATCGTGTTTCG**[G/A]**G[C/T]GCGTGAGGAGCTTACAAAAAAAATGCCAATAACTGGAATATGTGAAGTCATTGGATTCAACACTTTCTTCTAGCGATGGCACCAATGTTTGTAACTTGCAGGGAAAAGGAGGACATAGAACTGAACACTTGCTGATACTACTAACACAAGCAAAGATGTCTGTAAATATTGTTGTTCGATGACTAGTAAAATTTAAGTTGGAGAACATCTCAACCTATTACCCTGCCAGATACTGGGTTCCACACGCACACTGACATGATGTGGTGTTTACCTTTAGGTAGGGGAGTTTGTGGATAGATCGCCTAGCTCTATATAATATGCCGAGCGTCCGAGGTACCTGCTCAG

**Cyp6P9a- (350bp G>A)**

Wild-type allele – (G**G**G, 350bp) (**C**GC, 352bp)

Resistant allele – (G**A**G, 350bp) (**T**GC, 352bp)

**Primers:**

AfunCYP6P9a-F – 5’ CGGTGCACTTATGAACAAAGCTG 3’

AfunCYP6P9a-R – 5’ CGCTAGAAGAAAGTGTTGAATCC 3’

TM-F= 57°C, TM-R= 54°C

Product size= 173bp

**LNA Probes:**

CYP6P9a-AFSuS (HEX)- TTTC+G+GG+C+GC

CYP6P9a-AFRes (FAM)- TT+TC+G+AG+T+GCGT

Note:

CYP6P9a-AFSuS - (HEX)- (GGG, 350bp) is the Wild type (susceptible) allele CYP6P9a-AFRes - (FAM)- (GAG, 350bp) is the Mutant (resistant) allele.

**Protocol:**

PCR Mix: X1

2X Primetime/ Luna® Universal Probe qPCR Master Mix 5.00 µL (final conc. 1X)

Primer AfunCYP6P9a-F (10 µM) 0.35 µL (final conc. 0.35µM)

Primer AfunCYP6P9a-R (10 µM) 0.35 µL (final conc. 0.35µM)

Probe CYP6P9a-AFSuS (HEX) (10 µM) 0.20 µL (final conc. 0.20µM)

Probe CYP6P9a-AFRes (FAM) (10 µM) 0.20 µL (final conc. 0.20µM)

Molecular grade water 2.90 µL

Genomic DNA 1.00 µL

10.0 µL

PCR reaction conditions:

- 3 minutes denature at 95°C; (Segment 1)
- 20 cycles of denaturation for 15 seconds at 95°C, annealing for 30 seconds at 66°C; (Segment 2)
- 23 cycles of denaturation for 10 seconds at 95°C, annealing for 20 seconds at 58°C, and an extension of 10 seconds at 72°C (Segment 3)……………….as shown below.


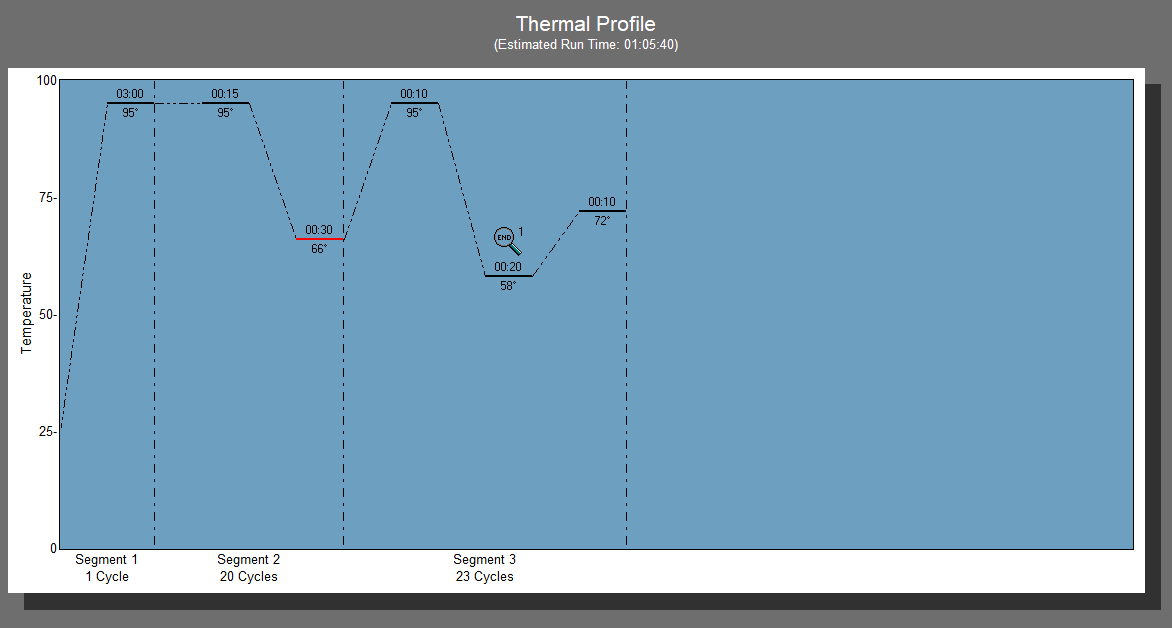


Analysis:

- Analyse the genotypes by looking at the fluorescence for RLast/RFirst. The FAM dye (Mutants) should be on the y-axis and HEX dye (wildtypes) on the x-axis as shown below.


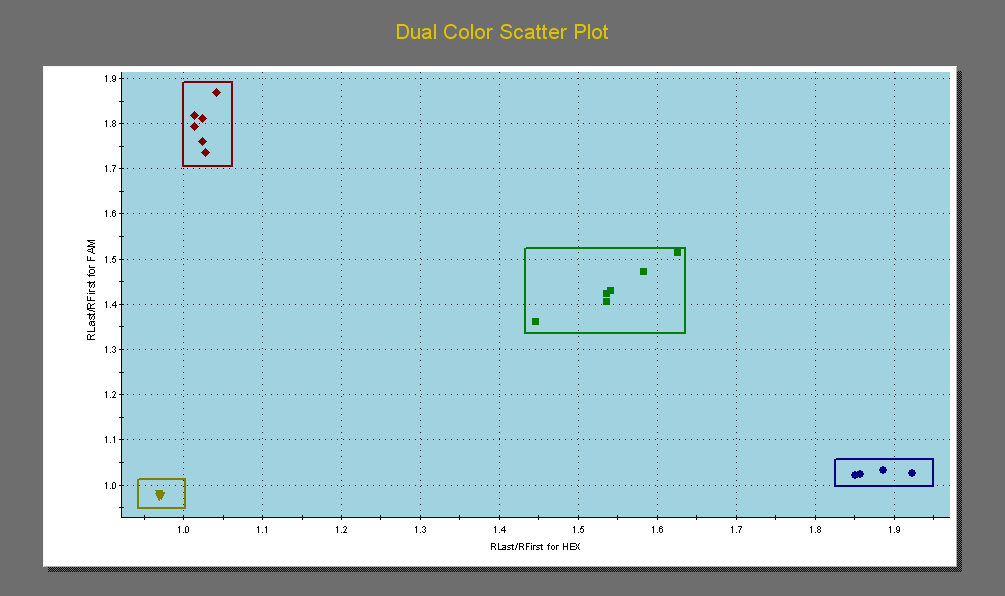


RR

RS

SS

NTC

1. **Protocol for genotyping the CYP6P9b marker in *An.funestus* using Locked Nucleic Acid (LNA) Assay**

Gene sequence:

GAATGGAAAGAACCGAAAGTGAAAAGGGCGTTCTTGTTGAAATATAATAGAGAATAAACATAAATACCTGTTGACACCCAAAATATCCTTTAATAACAAATGTGTTTTTTTCGTAATTTGTTTGAAGTCCTTGGTTGTAGGTAAGGTTAATATAAAGCAGGCTTAAACAAGTTGACCATTACGCCGAATAATGATCAAAAGGAAGAAACGATAGCAAGAGCTCCTCGACTTCTGCTCATGTGGGGCGCTTTAGTTGAGATTCGAACCAAGATCGGAAGTTTCTTTTGTCCAAGCCATAACAAATGTACCATG**[T/C]**GACC[T/A]TATATCTTA[A/C]AG[G/A]CACTAATAAAGTCAGTTTGATGCTGATTTTGTTGAATTATCCCAATTGGTAAATTTAAAATATCAATTGATTATAAATTCCATTTATTATGTACATTTGCAAGTGAGCGTGTAGTGGTGAAAAAATCCATCGCTATTGAGTTACGGATAATTAACTTAAATTAAATGTTCAAAGTCACCTGCACTTGAATCGTGTTTCGAGTGCGTGAGGAGTTTGCAAAAAACAATACACGTTTCTGGGATATGTGAAGTCATTGCATTCAACACTTTCTTCCAGCGGTATTTCAAATCTATGTAACTGTCTTTGAGAATAAAATCACATACTCATAATAACTAGATGCGTGTTGATACCAACACACAGGAAGATGTTTATGAATATTGTTGTTCGATGACTAGTAAAATTTATGTTGGAGACCATCCCAACCCTGTCAGATACTGGGTTCCACACGCACACTGACACGATGTGCTGATTACTCTTTGGTAGGCGAGTTTGTGGGTAGATCGCCCAGTTCTATATAATATGCCTAGCACCCATCGTATCTGCTCAGTTAGTTCGCGTGGCTTGAATTCGGCGAACGAAA

**Cyp6P9b- (313bp T>C)**

Wild-type allele – (G**T**G, 313bp)

Resistant allele – (G**C**G, 313bp)

**Primers:**

AfunCYP6P9b-F - 5’TTCGAACCAAGATCGGAAGTT 3’

AfunCYP6P9b-R – 5’TCAGCATCAAACTGACTTTATTAGTG 3’

TM-F= 54°C, TM-R= 54°C

Product size= 102bp

**LNA Probes:**

CYP6P9b-AFSuS (HEX)- CC+AT+G+T+GAC+C+T+TA

CYP6P9b-AFRes (FAM)- CAT+G+C+GAC+C+A+TA+TA

Note:

CYP6P9a-AFSuS - (HEX)- (GTG, 313bp) is the Wild type (susceptible) allele CYP6P9a-AFRes - (FAM)- (GCG, 313bp) is the Mutant (resistant) allele.

**Protocol:**

PCR Mix: X1

2X Primetime/ Luna® Universal Probe qPCR Master Mix 5.00 µL (final conc. 1X)

Primer AfunCYP6P9b-F (10 µM) 0.35 µL (final conc. 0.35µM)

Primer AfunCYP6P9b-R (10 µM) 0.35 µL (final conc. 0.35µM)

Probe CYP6P9b-AFSuS (HEX) (10 µM) 0.40 µL (final conc. 0.40µM)

Probe CYP6P9b-AFRes (FAM) (10 µM) 0.40 µL (final conc. 0.40µM)

Molecular grade water 2.50 µL

Genomic DNA 1.00 µL

10.0 µL

PCR reaction conditions:

- 3 Hot start denaturation at 95°C; (Segment 1)
- 40 cycles of denaturation for 30 seconds at 95°C, annealing for 30 seconds at 60°C (Segment 2) …………………………as shown below.

**
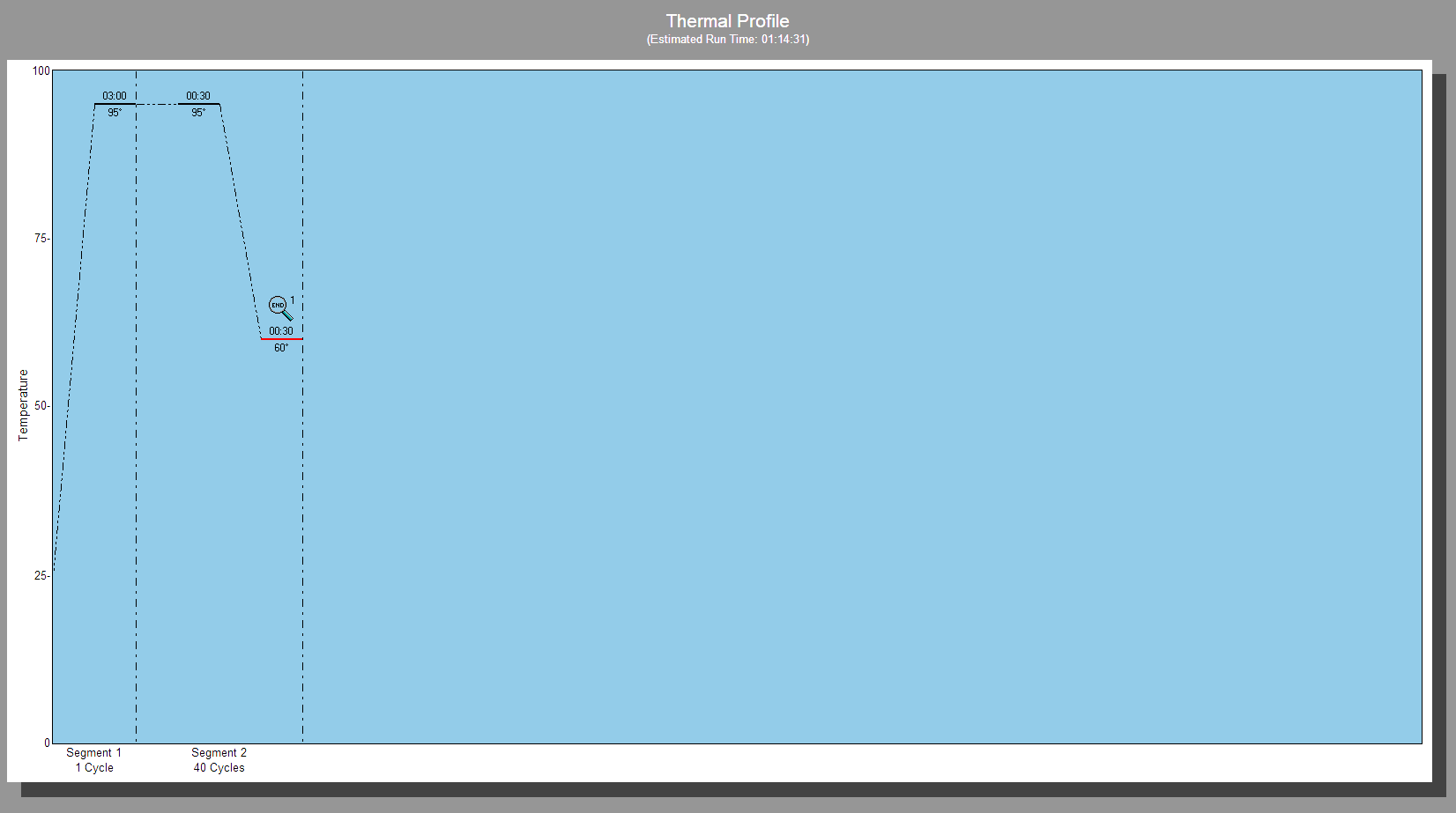
**

Analysis:

- Analyse the genotypes by looking at the fluorescence for dRLast for FAM (Mutants) and dRLast for HEX (Wildtypes) as shown below.


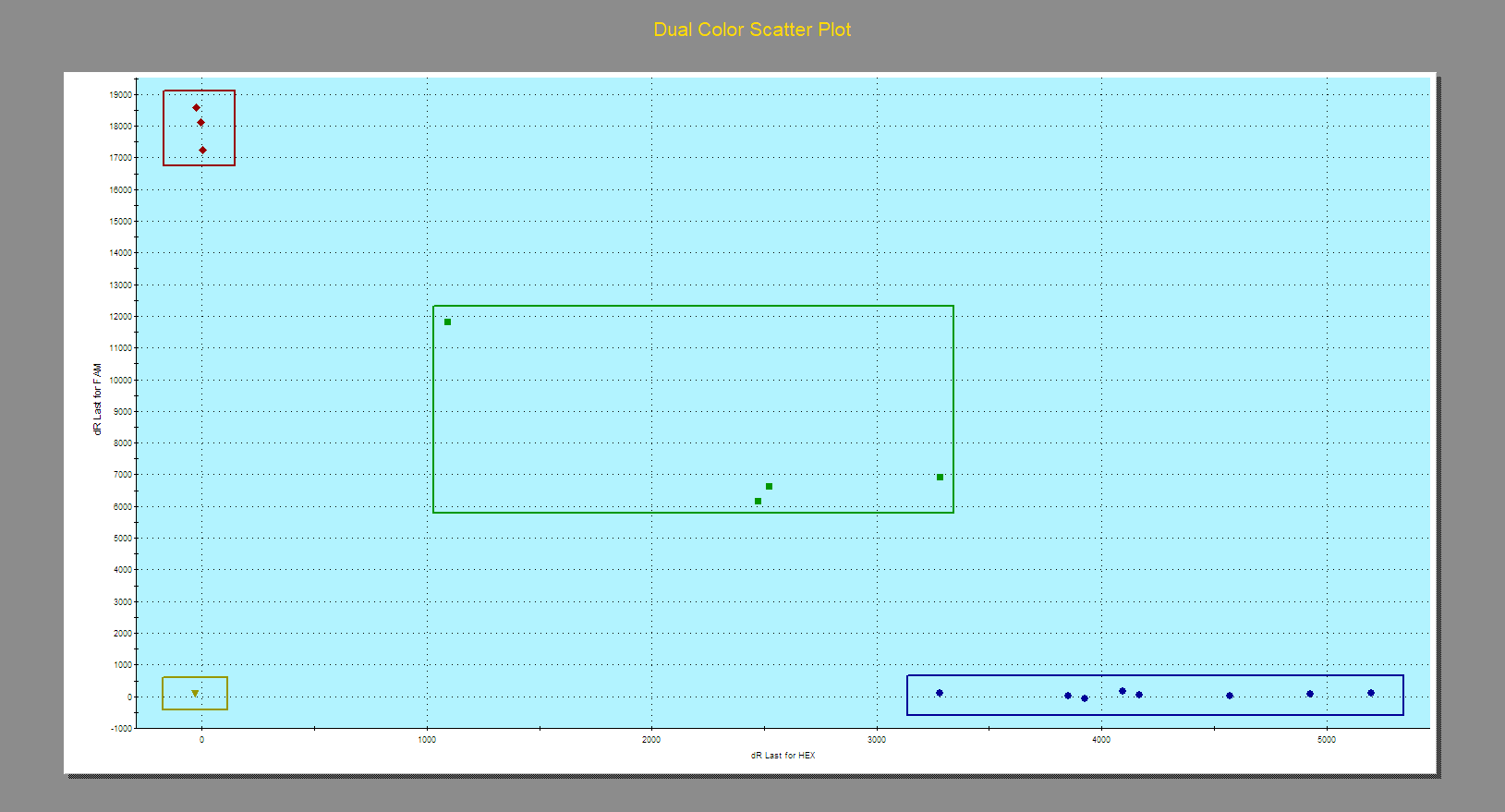


1. **Protocol for genotyping the GSTe2-L119F marker in *An.funestus* using Locked Nucleic Acid (LNA)Assay**

**Primers:**

Gste2-2F: 5’ ATGACACCCTGTACCCAAAAGATCC 3’

Gste2-2R: 5’ CTCCAGCAAGCGGTACGATTTC 3’

TM-2F= 58°C, TM-2R= 58°C

Product size= 182bp

**LNA Probes:**

Gste2-Leu (HEX)- 5’ AG+CGTAT+T+**C**+TT+TT+CT 3’

Gste2-Phe (FAM)- 5’ AG+CG+TAT+T+**T**+TTT+T+CT 3’

Note: Gste2-Leu (HEX)- (**C**TT, 119L) is the Wild type (susceptible) allele while Gste2-Phe (FAM)- (**T**TT, 119F) is the Mutant (resistant) allele.

**Protocol:**

PCR Mix: X1

2X Primetime/ Luna® Universal Probe qPCR Master Mix 5.00 µL (final conc. 1X)

Primer Gste2-2F (10 µM) 0.35 µL (final conc. 0.35µM)

Primer Gste2-2R (10 µM) 0.35 µL (final conc. 0.35µM)

Probe Gste2-Leu (HEX) (10 µM) 0.20 µL (final conc. 0.20µM)

Probe Gste2-Phe (FAM) (10 µM) 0.20 µL (final conc. 0.20µM)

Molecular grade water 2.90 µL

Genomic DNA 1.00 µL

10.0 µL

PCR reaction conditions:

- 3 minutes denature at 95°C; (Segment 1)
- 20 cycles of denaturation for 15 seconds at 95°C, annealing for 30 seconds at 66°C; (Segment 2)
- 23 cycles of denaturation for 10 seconds at 95°C, annealing for 20 seconds at 58°C, and an extension of 10 seconds at 72°C (Segment 3)……………….as shown below.


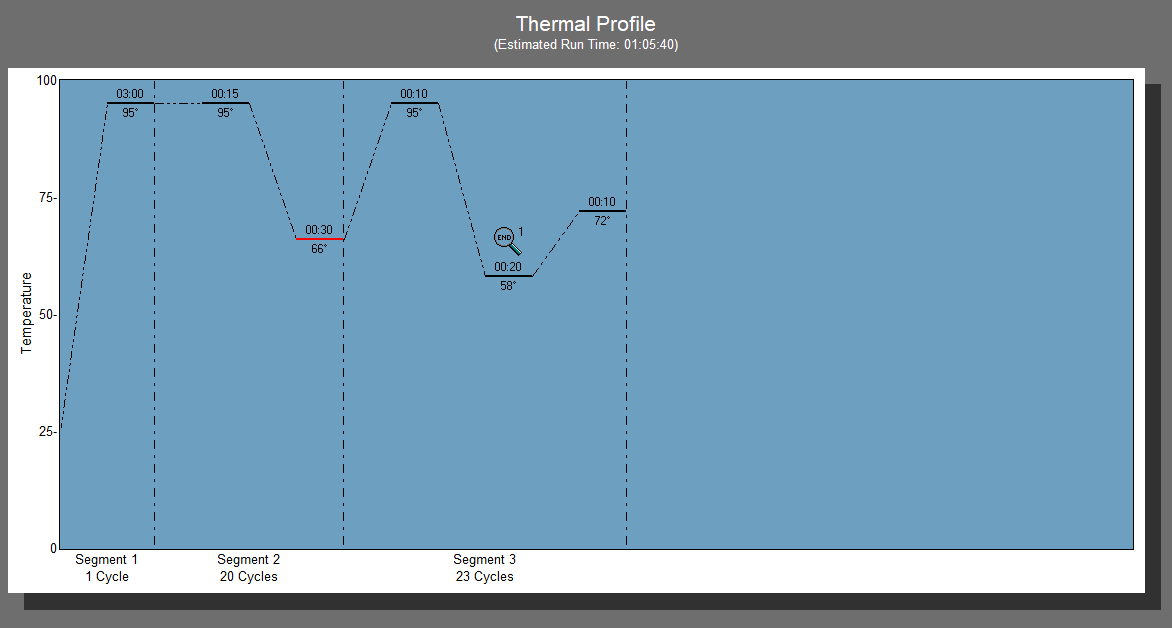


Analysis:

- Analyse the genotypes by looking at the fluorescence for RLast/RFirst. The FAM dye (Mutants) should be on the y-axis and HEX dye (wildtypes) on the x-axis as shown below.


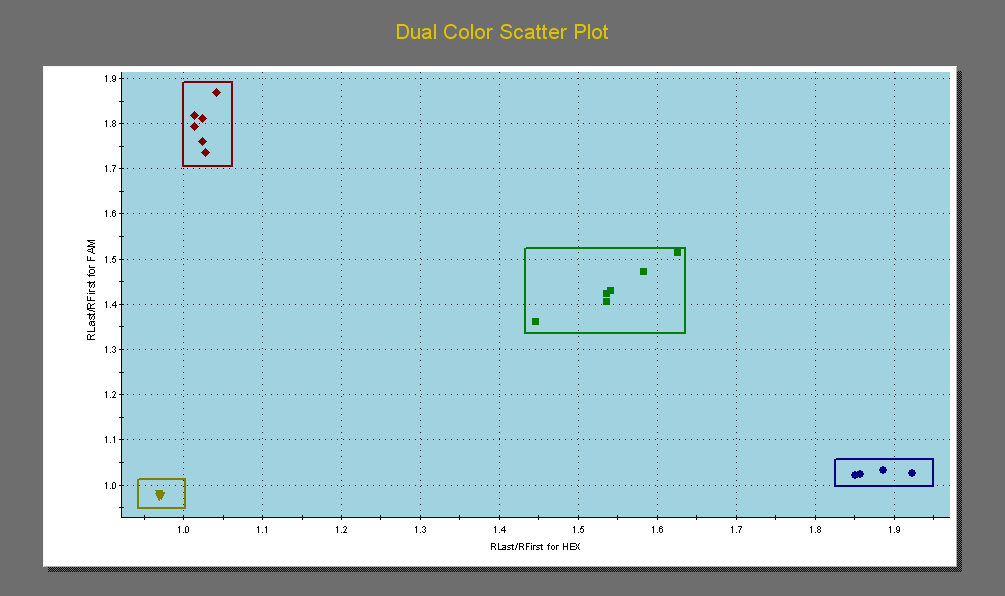


RR

RS

SS

NTC
